# Targeting lanosterol synthase alleviates MASLD by promoting fatty acid catabolism

**DOI:** 10.1007/s00018-026-06091-7

**Published:** 2026-03-17

**Authors:** Sumei Zhang, Mingcong Li, Xiaomei Sun, Dake Huang, Li Liu, Zhen Yang, Hongmei Bai, Weikang Hu, Wenjing Zhou, Zihan Wang, Jun Zhang, Zhenhai Tang, Sheng Wang, Qing Zhou, Yuan Wang, Yechuan Xu, Zhen Zhang, Ming Wang, Min Zhao, Shengquan Zhang

**Affiliations:** 1 Department of Biochemistry and Molecular Biology, School of Basic Medical Sciences, Anhui Medical University, Hefei, Anhui China; 2 Department of Pathology, Affiliated Hefei Hospital of Anhui Medical University, Hefei, Anhui China; 3 Comprehensive Laboratory in School of Basic Medical Sciences, Anhui Medical University, Hefei, Anhui China; 4 Center for Scientific Research of Anhui Medical University, Hefei, Anhui China; 5 Laboratory of Molecular Biology, Anhui Medical University, Hefei, Anhui China; 6 Department of General Surgery, The First Affiliated Hospital of Anhui Medical University, Hefei, Anhui China; 7 Division of Life Sciences and Medicine, University of Science and Technology of China, Hefei, Anhui China

**Keywords:** Methionine and choline-deficient diet, Ketogenesis, Fatty acid β-oxidation, Cholesterol

## Abstract

**Background & aims:**

Abnormal cholesterol metabolism is involved in the development of metabolic dysfunction-associated steatotic liver disease (MASLD). We investigated the role and mechanisms of lanosterol synthase (LSS) loss of function in the pathological process of MASLD.

**Methods:**

MASLD models were induced by methionine-and choline-deficient diet (MCD) feeding in LSS^+/−^ mice or wild type mice given LSS inhibitor RO48-8071. Transcriptomics analysis was performed to analyze differentially expressed genes in mice model. Lipidomic Profiling revealed the overall composition of lipid classes in MCD induced MASLD model. In vitro experiments using a MCDE (identical medium completely deficient of methionine and choline) induced cell model assessed the effect of LSS knockdown on MASLD development. Quantitative real-time PCR (qRT-PCR) and Western blot were employed to evaluate the differential expression of interested genes.

**Results:**

In MCD induced MASLD models, obviously reduced steatotic phenotype, hepatic inflammatory injury and hepatocyte ballooning were found in LSS^+/−^ mice. LSS loss of function alleviates liver injury and hepatic steatosis via reducing fatty deposition and triglyceride accumulation in hepatocytes. Mechanistically, LSS dysfunction promotes fatty acid β-oxidation and ketogenesis in liver cells to mediate attenuating effect on MASLD development.

**Conclusions:**

Targeting LSS alleviates MASLD development by promoting fatty acid β-oxidation and ketogenesis.

**Supplementary Information:**

The online version contains supplementary material available at 10.1007/s00018-026-06091-7.

## Introduction

 Metabolic dysfunction-associated steatotic liver disease (MASLD), termed as non-alcoholic fatty liver disease (NAFLD) before June 2023 [1], is emerging as a severe and major human-threatened disease worldwide. MASLD is a multifactorial chronic liver disease which will lead to steatohepatitis (MASH) and hepatitis. If not rationally and properly treated, some cases may potentially progress to fibrosis, cirrhosis and ultimately, hepatocellular carcinoma (HCC). The overall prevalence of MASLD worldwide is about 32.4%, with soaring prevalence in China from 25.4% in 2008 to 32.5% in 2019 [2]. Currently, only one pharmacological drug Resmetirom [3] was approved recently for the treatment of MASH with liver fibrosis, although numerous researchers have been working diligently to identify therapeutic targets for MASLD. The dramatic increase in the incidence and limited satisfactory treatment modalities of MASLD make it been challenging to understand the molecular mechanisms underlying the disorders.

Abnormal cholesterol metabolism is one of the main pathogenic factors for the occurrence and development of MASLD [4]. The mainstay drug for cholesterol lowering, statins, tempered MASLD development, but with great side effects. Because a variety of bioactive molecules downstream were reduced by inhibition of HMGCoA reductase, such as FPP (farnesyl pyrophosphate) and GGPP (geranylgeranyl pyrophosphate) [5]. Lanosterol synthase (LSS) catalyzes the production of the first cyclized intermediate lanosterol in the process of endogenous cholesterol synthesis and also the metabolic flux from epoxy squalene to 24, 25-epoxycholesterol [6]. Theoretically, LSS loss of function leads to reduction of sterols and cholesterol but no significant side effect caused by deprived or cumulative upstream intermediates. In addition, partial inhibition of LSS could increase 24(S), 25-epoxycholesterol production. 24(S), 25-epoxycholesterol attenuates foam cell formation by activating LXR signaling pathway in the process of MASLD developmentn [7]. Thus it could be speculated that LSS may be a more suitable therapeutic target for MASLD treatment.

The primary objective of this is to evaluate the role and mechanisms of LSS inhibition on MASLD development through in vitro and in vivo experiments. The molecular mechanisms of MASLD are extremely complex involving multiple biochemical processes happened in various organelles including mitochondrial energy metabolism [8]. By establishing a MASLD mouse model, we found that targeting LSS alleviates the development of MCD (methionine-and choline-deficient diet) induced MASLD in C57BL/6J mice with less severe manifestations of hepatic steatosis and inflammation. Less severe mitochondria damage and metabolically increased fatty acid catabolism such as fatty acid β-oxidation and ketogenesis were considered to be the possible mechanisms of mild hepatic steatosis and inflammation by targeting LSS. Our findings offer new insights for seeking therapeutic targets in MASLD therapy.

## Materials and methods

### Ethics statement

All patients involved in the study have provided informed consent for using the samples for research purposes after approved by the Institutional Review Board (IRB) of Anhui Medical University with approval no. 20,200,288. All mice studies and experimental procedures were conducted after approved by Institutional Animal Care and Use Committee of Anhui Medical University (Hefei, Anhui, China, permits 20201102). In conducting researches with mice, the investigators adhered to the strict guidelines and regulations of Institutional Animal Ethics Committee in Anhui Medical University.

## Human samples

Liver tissues of MASLD patients according to liver histology and healthy controls from the patients undergoing hepatic hemangioma resection were collected at the First Affiliated Hospital of Anhui Medical University. Liver histology by H&E staining was scored by pathologists according to NASH-CRN scoring system [9]. Cases were classified as MASLD with a MASLD activity score ≥ 3 and were treated as healthy controls with MASLD activity score of 0. Individuals with viral infection (hepatitis B or C virus), drug or toxin use, excessive alcohol consumption (for men, > 140 g/week; for women, > 70 g/week) or other decompensated liver diseases such as liver autoimmune were excluded from the present study [10].

## Generation of LSS knockout mice

LSS^+/−^ mice were prepared by Biomedical Research Institute of Nanjing University (Nanjing, China) using a pure C57BL/6 background. Global LSS knockout mice were acquired via CRISPR/Cas9 system [11] due to lethality of LSS homozygous knockout. Cas9 mRNA and sgRNA (5’-GAGCCGCCAGCGGGTGAGAT-3’ followed by the PAM sequence CGG) targeting LSS gene were co-injected into zygotes. sgRNA directed Cas9 endonuclease cleavage at exon 2 and created a double-strand break. Such breaks were repaired by non-homologous end joining and resulted in disruption of LSS gene with 46 bps deletion by frame shift from exon 2. The microinjected zygotes were transplanted into pseudopregnant mice. The genomic DNA of the newborn F0 mice was extracted for sequencing using primers flanking the target site. Transgenic positive founder mice were mated with wild-type mice to generate positive F1 mice and the pups were genotyped by PCR followed by sequence analysis. Knockout and WT genotypes were verified with PCR analysis of tail clips using the primers forward ACCTGGGCGGAGTCTAAGGAAG and reverse AAGGCTCTCCACTGTTTCAGAGCT.

### Animal models

 At 8 weeks of age, LSS^+/−^ mice and their non-transgenic littermates were fed MCD diet [10] (Research Diets, NJ, Supplementary Table 3) for 2 weeks with parallel control mice fed a normal chow diet (ND, Research Diets, NJ). The groups are as follow (*N* = 10 for each group): ND-fed WT mice, MCD-fed WT mice, ND-fed LSS^+/−^ mice, and MCD-fed LSS^+/−^ mice. Male eight-week-old WT mice were fed with ND or MCD diet for two weeks, with or without intraperitoneal injection of LSS inhibitor, RO48-8071 (MedChemExpress, NJ), at 10 mg/kg/day to confirm the role of LSS loss of function in MCD-induced liver injury model, with PBS serving as control.

Body weight (BW) of each mice was recorded weekly during the period of feeding. Retro-orbital blood samples were collected and serum was separated and stored at − 80 °C. Liver samples were collected snap frozen, or fixed in 4% formaldehyde solution or 3% glutaraldehyde for further experiments when the mice were sacrificed. Mice and livers were weighed and the pictures of livers were captured. The liver index of each mouse was calculated as liver wet weight/body weight × 100%.

## Cell models

### Construction of LSS knockdown cell line

HepG2 cells were transfected with 500 ng confirmed shLSS-pRNAT-U6.1/Neo targeting human LSS gene sequences GGACTGCGCTCAACTATGT or pRNAT-U6.1/Neo vector plasmids using Lipofectamine 2000 (11668019, Thermo Fisher Scientific, Waltham, MA, USA) and selected with G418 (HY-17561, MedChemExpress, NJ, USA) to get stably transfected HepG2 cell clones. The efficiency of transfection and RNA interference by shRNAs were verified by green fluorescence and LSS expression level, respectively.

#### Methionine and choline-deficient cell model

HepG2 cell clones with LSS knockdown by shRNA were exposed to identical medium completely deficient of methionine and choline (MCDE, Thermo Fisher Scientific, Waltham, MA) and subjected Oil Red O and H&E staining together with primary hepatocytes isolated from LSS^+/−^ mice or WT mice fed MCD diet, or harvested for Western blot analyses.

### Plasma biochemistry analysis

The plasma levels of alanine aminotransferase (ALT), aspartate aminotransferase (AST), triglyceride (TG), total cholesterol (Tc), high density lipoprotein cholesterol (HDLc), low density lipoprotein cholesterol (LDLc) and glucose (GLU) were measured using HITACHI3100 automatic biochemical analyzer (HITACHI3100, Tokyo, Japan) according to the manufacturer’s instructions.

## Analysis of lipid components in the liver

Liver tissues were homogenized and lysed with the enzymatic kit (GPO-POD, Applygen Technologies, Beijing) to get extracts for analysis of total TG, Tc, HDLc and LDLc using HITACHI 3100 automatic biochemical analyzer. And the differences in lipid metabolites among the groups were quantitatively analyzed using ultra-high performance liquid chromatography (UHPLC) Dionex Ultimate 3000 system (Thermo Fisher Scientific, San Jose, USA).

## Histology staining

### H&E staining for general observation

Fresh liver tissues were fixed in 10% formalin for 48 h, dehydrated, embedded in paraffin and sliced into 4 μm sections (HistoCore AUTOCUT, Leica RM2255, Germany). H&E staining for tissue slices and cells fixed in 10% formalin were performed using a standard protocol.

### Oil red O staining for intracellular lipid droplets observation

Fresh liver tissues were snap frozen in liquid nitrogen and embedded in optimal cutting temperature compound (OCT) embedding medium, and then sliced into 8 μm thick sections on a frozen section machine (Cryostat microtome, Leica CM3050S, Germany). Oil Red O staining was performed to assess lipid accumulation in hepatocytes and treated HepG2 cells. Lipid accumulation in liver tissues and cells was quantified via the amount of Oil Red O staining using Image J Software.

### Sirius red staining for collagen fiber detection

Paraffin-embedded histology slides were stained using Sirius Red in saturated picric acid (Sigma Aldrich, St Louis, MO) and pictured to observe collagen fibers.

All histological slides were visualized on TissueFAXS Plus (version 7.1, TissueGnostics GmbH, Vienna, Austria). Pathologic features of the liver sections were graded in a blinded fashion following the scoring system by Kleiner et al. [13] to assess the severity of MASLD. The average score of histological characteristic in each group was presented.

### Ultrastructural analysis by electronic microscopy

Fresh liver tissues were embedded in Epon 812 (Ted Pella, CA). Ultrathin sections were cut at 70 nm and double-stained with uranyl acetate and lead citrate, and photographed by transmission electron microscope (Talos L120C G2; Thermo Fisher Scientific).

### Quantitative transcriptome analysis of liver tissues

Total RNA extracted from fresh liver tissues was used as template to synthesize cDNA and cyclized to obtain a single strand circular DNA library which is replicated through rolling rings to form DNA nanoball (DNB) and sequenced through combinatorial Probe-Anchor Synthesis (cPAS) (BGI-Shenzhen).

### Array data and pathway analysis

GO (http://www.geneontology.org/) and Kyoto Encyclopedia of Genes and Genomes (KEGG, https://www.kegg.jp/) enrichment analysis of annotated different expression gene was performed by Phyper based on Hypergeometric test.

### Validation of target genes using qRT-PCR analysis

Quantitative real-time PCR (qRT-PCR) was done to evaluate and confirm their expression levels of interested genes which were shown differently expressed between groups in the array results. The primers used for target genes are listed in Supplementary Table 1.

### Western blot analysis

Equal amounts of heat-denatured protein extracted from snap frozen liver tissues or treated cells was subjected to SDS-PAGE, transferred onto polyvinylidene fluoride (PVDF) membrane (Millipore, Billerica, MA) and probed with primary antibodies and horseradish peroxidase-conjugated secondary antibodies sequentially. Chemiluminescence signals were developed by a chemiluminescent imaging instrument (14T12NPFLI6-348; Tanon) using an ECL kit (Thermo Fisher Scientific, Waltham, MA). The grayscale of each objective band was determined using Image J Software and the densitometric analysis of all Western blots bands were shown as relative intensity to that of Beta-actin. The antibodies used are listed in Supplementary Table 2.

### Statistical analysis

SPSS and GraphPad Software were used for all the statistical tests in the present study. All the data were expressed as means ± Standard error of the mean (SEM). The differences among the groups were analyzed using One-way analysis of variance (ANOVA) and least significant difference (LSD). Threshold for statistical significance was *p* < 0.05.

Detailed methods are provided as Supplementary Materials and Methods.

## Results

### Heterozygous knockout of LSS is efficient

**Fig. 1:**
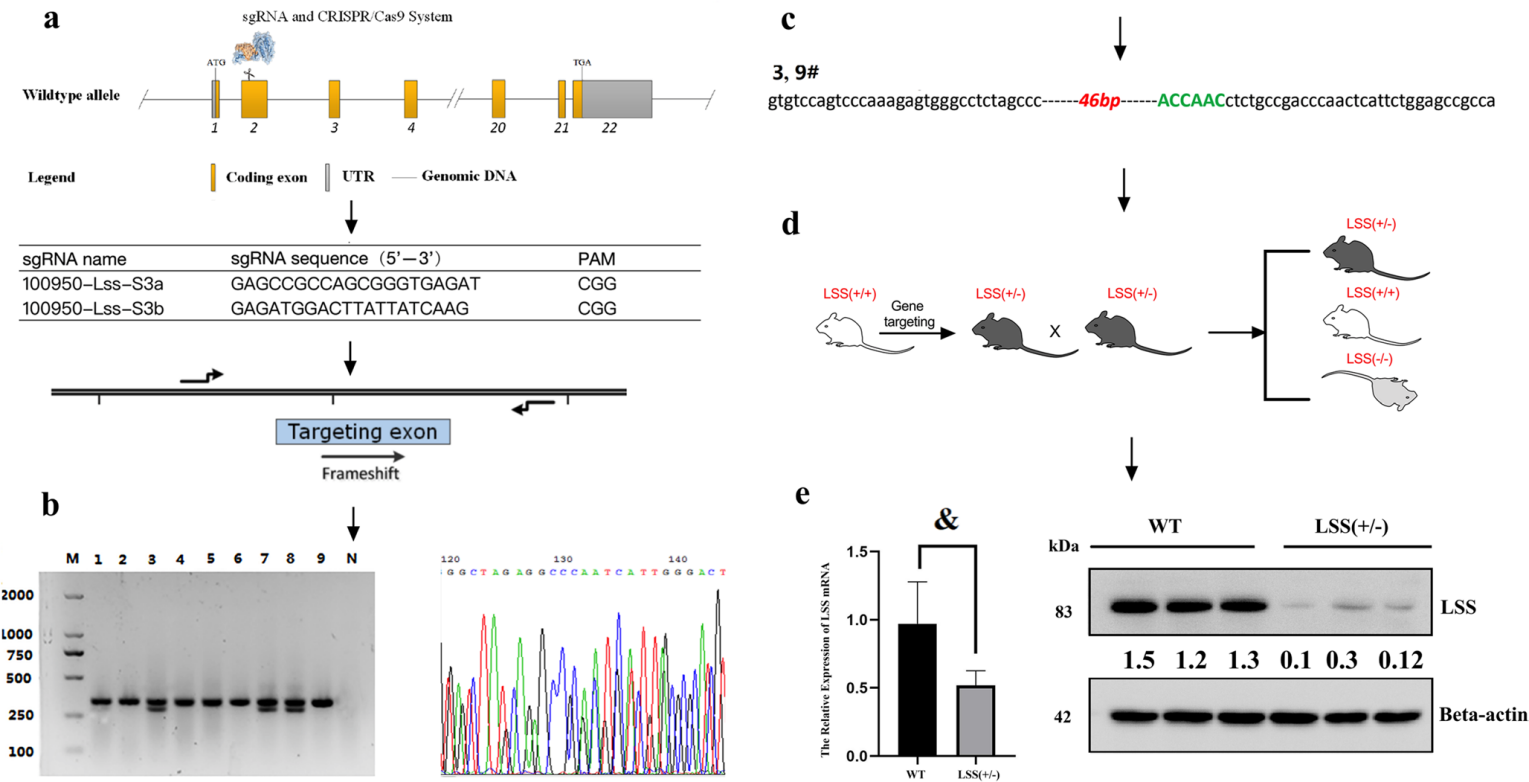
Heterozygous knockout of LSS was efficiency. (**a**) Strategy for heterozygous knockout of LSS by CRISPR Cas9, (**b**) genotype identification by PCR and DNA sequencing, (**c**) LSS gene sequence after specific knockout, (**d**) crossing pattern. (**e**) LSS is effectively knocked down identified by qRT-PCR and Western blot of liver lysate. ^&^*p* < 0.01

LSS heterozygous knockout mice, due to lethality of LSS homozygous knockout, were established using a pure C57BL/6 background via CRISPR/Cas9 system to generate a disruption of LSS gene with 46bps deletion by frameshift from exon 2 (Fig. 1). The genotype was identified by PCR with two bands at 328 bp and 282 bp of PCR products from LSS knockout mouse differentiated from wild type mouse (WT) with a single band at 328 bp. An evidently reduced LSS expression was confirmed by qRT-PCR and Western blot in liver tissues of LSS knockout mice. It could be concluded that heterozygous knockout of LSS is successful and efficient. Thus the mice with heterozygous LSS knockout were used in the present study and referred as LSS^+/−^ hereinafter.

### Functional inhibition of LSS delays pathological progression of MASLD in mice models

**Fig. 2:**
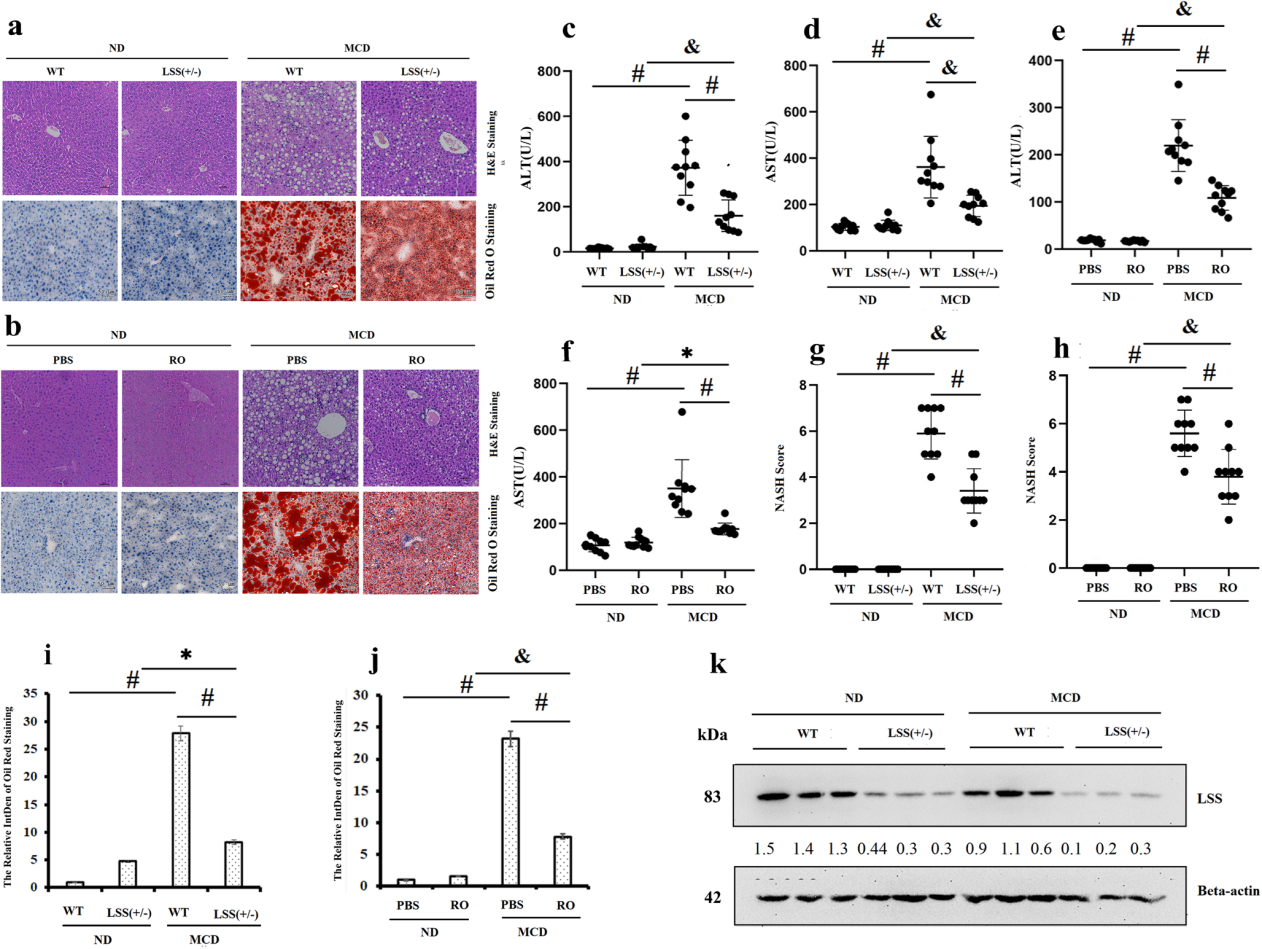
Heterozygous knockout of LSS alleviated MCD induced MASLD. (**a**) Liver H&E and Oil Red O staining of ND and MCD-fed WT and LSS^+/−^ mice, (**b**) Liver H&E and Oil Red O staining of ND and MCD-fed WT mice with or without RO 48–8071 injection, (**c**) Plasma ALT and (**d**) Plasma AST of ND and MCD-fed WT and LSS^+/−^ mice, (**e**) Plasma ALT and (**f**) Plasma AST of ND and MCD-fed WT mice with or without RO 48–8071 injection, (**g**) NASH score of ND and MCD-fed WT and LSS^+/−^ mice, (**h**) NASH score of ND and MCD-fed WT mice with or without RO 48–8071 injection, (**i**) Quantification of liver Oil Red O staining of ND and MCD-fed WT and LSS^+/−^ mice, (**j**) Quantification of liver Oil Red O staining of ND and MCD-fed WT mice with or without RO 48–8071 injection, (**k**) Liver LSS protein levels of WT and LSS^+/−^ mice fed ND and MCD. *N* = 10 mice for each group. ^*^p < 0.05, ^&^*p* < 0.01, ^#^*p* < 0.001

MCD diet was used to feed mice for two weeks to generate MASLD mice model [10]. Abnormal high serum ALT (Fig. 2c) and AST (Fig. 2d), steatotic phenotype presented by dispersed inflammation, hepatocyte ballooning and intracellular lipid droplets (Fig. 2a and i) without obvious fibrosis displayed typical features of MASLD (Supplementary Fig. 2) [14]. The liver histology and the serum marker level mentioned above show that the liver injury mice model was established successfully with hepatic steatosis.

Significantly alleviated pathological changes with lower serum ALT/AST level (Fig. 2c and d), less inflammatory cells infiltration and smaller lipid droplets (Fig. 2a and i) quantified as lower NASH score (Fig. 2g) were presented in hepatocytes from LSS^+/-^ mice, which suggests that the formation of larger lipid droplets may be delayed in the LSS^+/-^ livers under conditions of MCD feeding. The tentative experiment shown above revealed that hepatic damage and steatosis were reduced in LSS^+/-^ mice in comparison with that in WT mice. These give us a clue that LSS loss of function by heterozygous knockout protects mice from MCD-induced MASLD phenotype such as steatosis and inflammation signaling.

To confirm if LSS loss of function do play alleviating effect on the development of MASLD, specific inhibitor of LSS, RO48-8071, was intraperitoneally injected into the MCD-induced MASLD mice model. Consistent with the observations in LSS^+/−^ mice, alleviated pathological changes presented by lower serum ALT/AST (Fig. 2e and f) and less steatotic (Fig. 2b, h and j) were also observed in MCD-fed WT mice with simultaneous administration of RO48-8071, suggesting a role of RO48-8071 in preventing and recovering from MCD induced hepatic steatosis. The changes mentioned above, in LSS^+/−^ mice and RO48-8071 treated mice, both indicate the protective effects on MASLD by targeting LSS.

What should be mentioned is, no obvious difference could be detected between ND-fed WT and LSS^+/-^ mice, which excludes the possibility that LSS^+/-^ could lead to pathological changes in mice liver.

### Heterozygous knockout of LSS alter the overall composition of lipid classes in MCD MASLD model

**Fig. 3:**
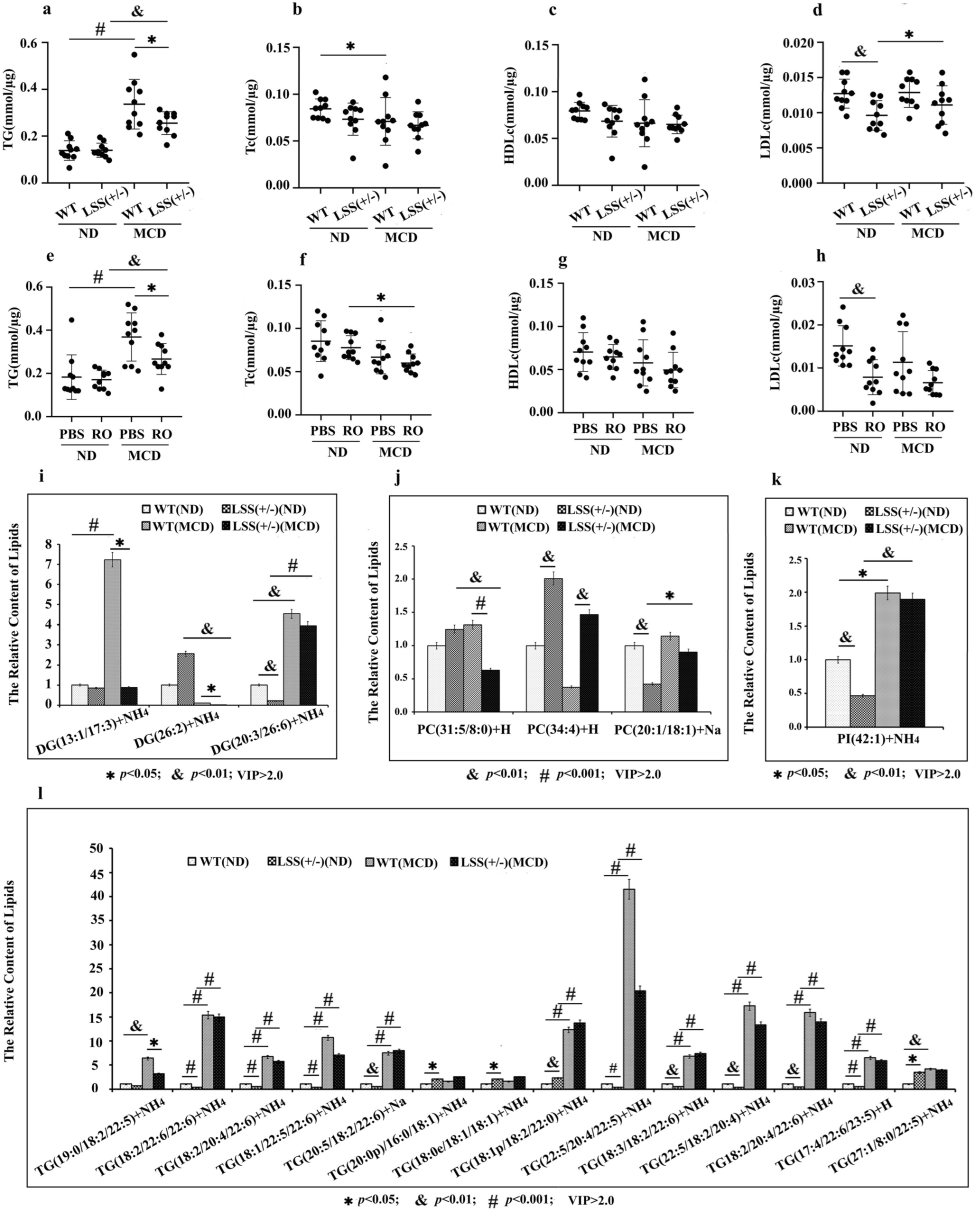
Heterozygous knockout of LSS alter the overall composition of lipid classes in MCD mouse model. (**a**) Hepatic TG, (**b**) Tc, (**c**) HDLc and (**d**) LDLc of ND and MCD-fed WT and LSS^+/−^ mice. (**e**) Hepatic TG, (**f**) Tc, (**g**) HDLc and (**h**) LDLc of of ND and MCD-fed WT mice with or without RO 48–8071 injection. (**i**) The relative contents of DGs, (**j**) PCs, (**k**) PIs, and (**l**) TGs in livers of ND and MCD -fed WT and LSS^+/−^ mice. *N* = 10 mice for each group. ^*^p < 0.05, ^&^*p* < 0.01, ^#^*p* < 0.001

The lipidomic analysis of liver tissues in mice detected that heterozygous knockout of LSS alter the overall composition of lipid classes in MCD mice model. MCD-fed WT mice showed higher TG and diacylglycerol (DG) than ND-fed ones which proved that MCD induced fatty liver successfully. When compare MCD-fed LSS^+/−^ mice to MCD-fed WT mice, liver TG and DG were much lower (Fig. 3a and i). ** (**Table 1**)**.

**Table 1:** The differential lipid components

	FC(MCD WT/LSS^+/−^)	*p*-VALUE	VIP
DG(13:1/17:3) + NH4	8.252959	0.017964	3.64071
DG(26:2) + NH4	4.997591	0.044756	3.35296
PC(31:5/8:0) + H	2.086874	0.000725	2.47368
TG(19:0/18:2/22:5) + NH4	2.022191	0.025471	2.07046
PC(34:4) + H	0.25628	0.003563	2.7693
	**FC(ND WT/LSS**^**+/−**^**)**	**p-VALUE**	**VIP**
DG(20:3/22:6) + NH4	4.771165	0.006075	3.20466
DG(26:2) + NH4	0.391287	0.034097	2.2464
PC(20:1/18:1) + Na	2.36986	0.002333	2.6858
PC(34:4) + H	0.497558	0.003356	2.21485
PI(42:1) + NH4	2.149056	5.61E-05	2.39292
TG(18:2/22:6/22:6) + NH4	3.527176	0.000178	3.0899
TG(22:5/18:2/20:4) + NH4	3.151646	0.001236	2.88909
TG(18:1/22:5/22:6) + NH4	3.099715	0.000943	2.82606
TG(22:5/20:4/22:5) + NH4	3.020072	0.000434	2.85556
TG(18:2/20:4/22:6) + NH4	2.513951	0.001266	2.53127
TG(18:3/18:2/22:6) + NH4	2.250835	0.00931	2.06565
TG(20:5/18:2/22:6) + Na	2.240542	0.00805	2.11825
TG(18:2/20:4/22:6) + Na	2.219078	7.62E-05	2.49098
TG(17:4/22:6/23:5) + H	2.19437	0.000768	2.41549
TG(18:0e/18:1/18:1) + NH4	0.496378	0.013245	2.20292
TG(20:0p/16:0/18:1) + NH4	0.493324	0.011575	2.23504
TG(18:1p/18:2/22:0) + NH4	0.438774	0.002907	2.50358
TG(27:1/8:0/22:5) + NH4	0.28908	0.049504	2.66276

Although phosphatidylcholine (PCs) contents were found to be changed in the mice model (Fig. 3j), but it is hard to discuss the role of PC because choline deficient diet was used in the present study, even if PC plays critical role in human disease including MASLD [16]. Similarly, TG levels in the mice livers of RO48-8071 treated MCD models were lower than that in PBS injection ones (Fig. 3e). In view of the above, we speculated that LSS inhibitor and LSS^+/−^ could both alleviate steatosis and thus ameliorates MASLD caused by MCD by reducing the accumulation of lipid components in liver, especially TG, as excess fatty acids are substrates for lipotoxic species to provoke hepatocellular injury [17].

### Numerous genes are differentially expressed in liver tissues

**Fig. 4:**
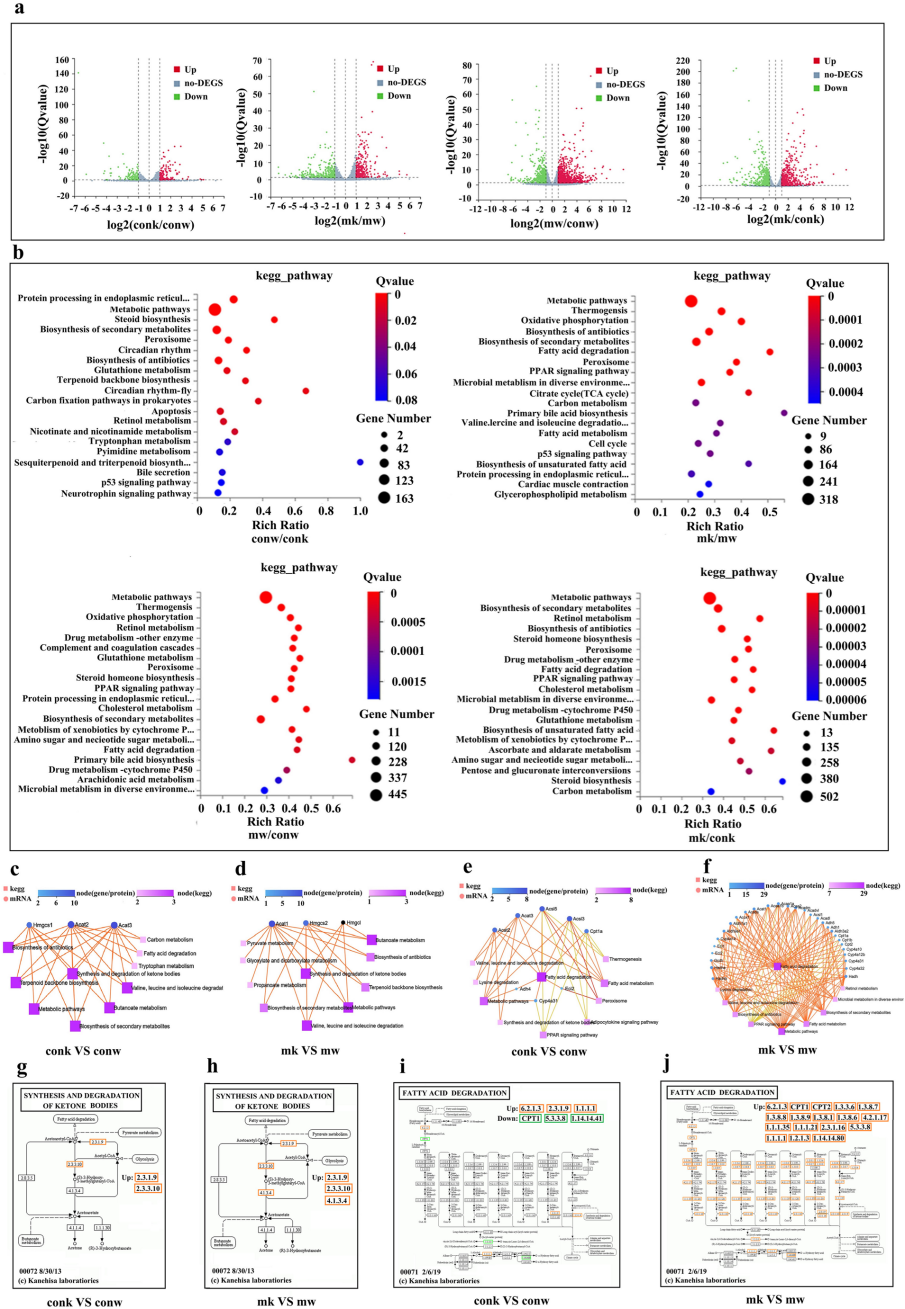
Heterozygous knockout of LSS changed the expression of numerous genes in mice livers. (**a**) Volcano plot of RNAseq, (**b**) Pathway enrichment analysis of RNAseq, (**c**) Differentially expressed genes involved in ketone bodies metabolism and associated pathways between ND-fed WT and LSS^+/−^ mice, and (**d**) between MCD-fed WT and LSS^+/−^ mice. (**e**) Differentially expressed genes involved in fatty acid degradation and associated pathways between ND-fed WT and LSS^+/−^ mice, and (**f**) between MCD-fed WT and LSS^+/−^ mice. (**g**) Location of differentially expressed genes between ND-fed WT and LSS^+/−^ mice, and (**h**) between MCD-fed WT and LSS^+/−^ mice in the metabolic process of ketone bodies synthesis and degradation. (**i**) Location of differentially expressed genes between ND-fed WT and LSS^+/−^ mice, and (**i**) between MCD-fed WT and LSS^+/−^ mice in the metabolic process of fatty acid degradation. *N* = 3 mice for each group. CONW: ND-fed WT mice, CONK: ND-fed WT and LSS^+/−^ mice, MW: MCD-fed WT mice, MK: M CD-fed LSS^+/−^ mice

Quantitative Transcriptome analysis of liver mRNA was performed to characterize the molecular targets involved in the protective role of LSS loss of function in MASLD development Transcriptome analysis implied altered expression of a large amount of genes and the relative expression of altered genes from the RNA-seq dataset between groups are shown as Volcano Plot in Fig. 4a. Totally, we found 1890 differentially expressed genes when compared the gene expression profiles of the livers from MCD-fed LSS^+/−^ and wild type mice (Only genes with Q value < 0.05 are displayed).

Functional enrichment analyses using KEGG pathways revealed that differentially expressed genes between MCD-fed LSS^+/−^ and WT mice are involved in multiple pathways (Fig. 4b) including Metabolic pathways, Thermogenesis, Oxidation phosphorylation, Biosynthesis of antibiotics, Fatty acid degradation, Peroxisome and Citrate cycle, etc. Many pathways mentioned above were related to lipid metabolism such as Thermogenesis, Oxidation phosphorylation, fatty acid degradation and Citrate cycle. The above results from quantitative transcripome analysis indicated that lots of lipid metabolism associated genes were involved in the protective role of LSS^+/−^ on MCD-induced liver injury.

### Heterozygous knockout of LSS activates β-oxidation and ketonebodies synthesis in MASLD mouse models

**Fig. 5:**
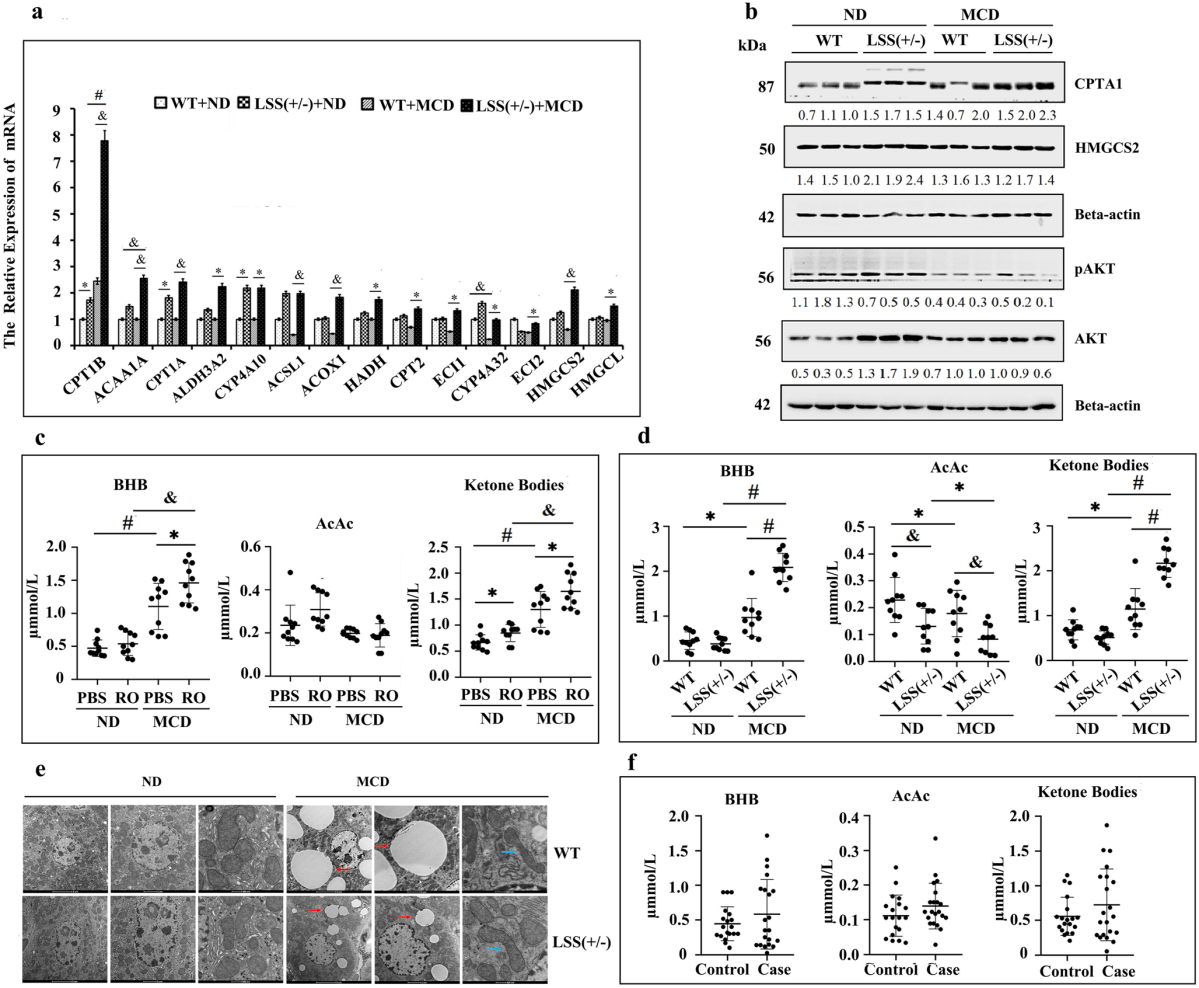
Heterozygous knockout of LSS activated β-oxidation and ketonebodies synthesis in MASLD mice models. (**a**) Liver mRNA levels of interested genes involved in ketogenesis and fatty acid catabolism of ND and MCD-fed WT and LSS^+/−^ mice. (**b**) Expression level of fatty acid β-oxidation and ketogenesis key enzymes and activities of AKT signaling pathway in liver lysates of ND and MCD-fed WT and LSS^+/−^ mice, (**c**) Serum ketone bodies levels of ND and MCD-fed WT mice with or without RO48-8071 injection, (**d**) Serum ketone bodies levels of ND and MCD-fed WT and LSS^+/−^ mice, (**e**) Liver mitochondrial ultrastructures of ND and MCD-fed WT and LSS^+/−^ mice (The arrows point to pathologic structural features), (**f**) Serum ketone bodies levels of MASLD patients and control. *N* = 10 mice for each group. ^*^p < 0.05, ^&^*p* < 0.01, ^#^*p* < 0.001

We next turned our attention to the involvement of lipid metabolism associated genes on the protective effect LSS inhibition on MASLD development. As detected by the lipidomic analysis TG levels in the livers of MCD-fed LSS^+/-^ were lower than that in WT ones Fatty acids in the liver are either esterified into TGs or metabolized by β-oxidation. TG homeostasis in hepatocytes is regulated by a complex set of mechanisms that include not only lipid biosynthesis but also lipid catabolism [18]. Based on transcriptome analysis and KEGG classification, differential gene expression profiles for lipid catabolism between MCD-fed WT and LSS^+/-^ mice were focused on fatty acid degradation with ketone bodies synthesis pathway included (Tables 2 and 3; Fig. 4f and j). Quantitative PCR confirmed differential expression of genes involved in fatty acid degradation and ketone bodies synthesis (Fig. 5a). Increased mRNA and protein level of ketogenesis gene HMGCS2 (HMG-CoA synthase family member), a mitochondrial enzyme that catalyzes the first reaction of ketogenesis [19] were found in livers of mice with heterozygous LSS knockout (Fig. 5a and b). Upregulated CTP1A (carnitinepalmitoyltransferase I), the key enzyme of fatty acid β-oxidation in the carnitine-dependent transport of acyl coenzyme A across the mitochondrial inner membrane [20], was also detected in LSS^+/-^ mice (Fig. 5a and b). Meanwhile, significantly increased serum β-hydroxybutyric acid (BHB) and total ketone bodies levels were detected in MCD-fed LSS^+/-^ mice when compared to MCD-fed WT mice (Fig. 5d). But no difference of BHB, acetoacetic acid (AcAc) or total ketone bodies levels was detected in MASLD patients when compared to healthy controls (Fig. 5f). Together with the similar finding in MCD-fed WT mice treated with RO48-8071 (Fig. 5c), these might suggest that fatty acid content was metabolized to generate acetyl-CoA through β-oxidation and then synthesize ketone bodies to be exported from the liver in the cases with LSS inhibition.

**Table 2:** Differentially expressed genes by KEGG pathway enrichment (fatty acid degradation)

Gene ID	Gene Symbol	log2 (conk/conw)	Qvalue (conk/conw)
74,205	ACSLc3	1.197538526	9.63E-11
666,168	CYP4A31	−1.204353651	1.29E-05
Gene ID	**Gene Symbol**	**log2 (mk/mw)**	**Qvalue (mk/mw)**
13,117	CYP4A10	2.600664974	4.34E-69
13,119	CYP4A14	2.519793489	3.85E-40
235,674	ACAA1B	2.155261238	4.46E-27
11,430	ACOX1	2.031848707	5.84E-21
14,081	ACSL1	1.967067362	2.02E-08
13,118	CYP4A12B	1.815169234	0.035817482
666,168	CYP4A31	1.518504422	1.67E-21
12,895	CPT1B	1.288789439	2.72E-05
13,177	ECI1	1.262680314	1.41E-09
11,522	ADH1	1.181447873	3.51E-04
11,671	ALDH3A2	1.138545555	2.12E-05
113,868	ACAA1A	1.072950688	1.91E-06
23,986	ECI2	1.042591666	2.26E-07
100,040,843	CYP4A32	1.00978674	5.27E-04
110,695	ALDH7A1	−1.147858683	3.39E-23
26,876	ADH4	−1.75798967	9.82E-35
13,118	CYP4A12B	−4.865050784	1.66E-20
277,753	CYP4A12A	−5.666817685	2.85E-25

**Table 3:** Differentially expressed genes by KEGG pathway enrichment (ketone bodies metabolism)

Gene ID	Gene Symbol	log2 (conk/conw)	Qvalue (conk/conw)
208,715	HMGCS1	1.91429897	1.25E-26
Gene ID	**Gene Symbol**	**log2 (mk/mw)**	**Qvalue (mk/mw)**
15,360	HMGCS2	1.086189428	4.90E-09

Mitochondrion is a key organelle for energy production and cellular adaptive response to various intracellular and environmental stresses [22]. Structural abnormalities of liver mitochondria were presented in patients and animal models of MASLD [23]. Although livers from WT and LSS^+/-^ mice both appeared phenotypically normal in the absence of challenge, ultrastructural analysis showed that after MCD feeding, comparatively mild hepatocyte mitochondrial injury was observed in LSS^+/-^ mice as relatively intact mitochondrial membrane structure with clearer and contacter mitochondrial crista, smaller lipid droplets and no obvious vacuolization when compared with the littermate control (Fig. 5e). This is consistent with the increased fatty acids β-oxidation and ketone bodies synthesis in hepacytes of LSS^+/-^ mice, both suggesting a stronger mitochondrial metabolic function than corresponding WT mice.

### LSS loss of function promoted β-oxidation and ketonebodies synthesis to alleviate MCD induced steatosis in cell models

**Fig. 6:**
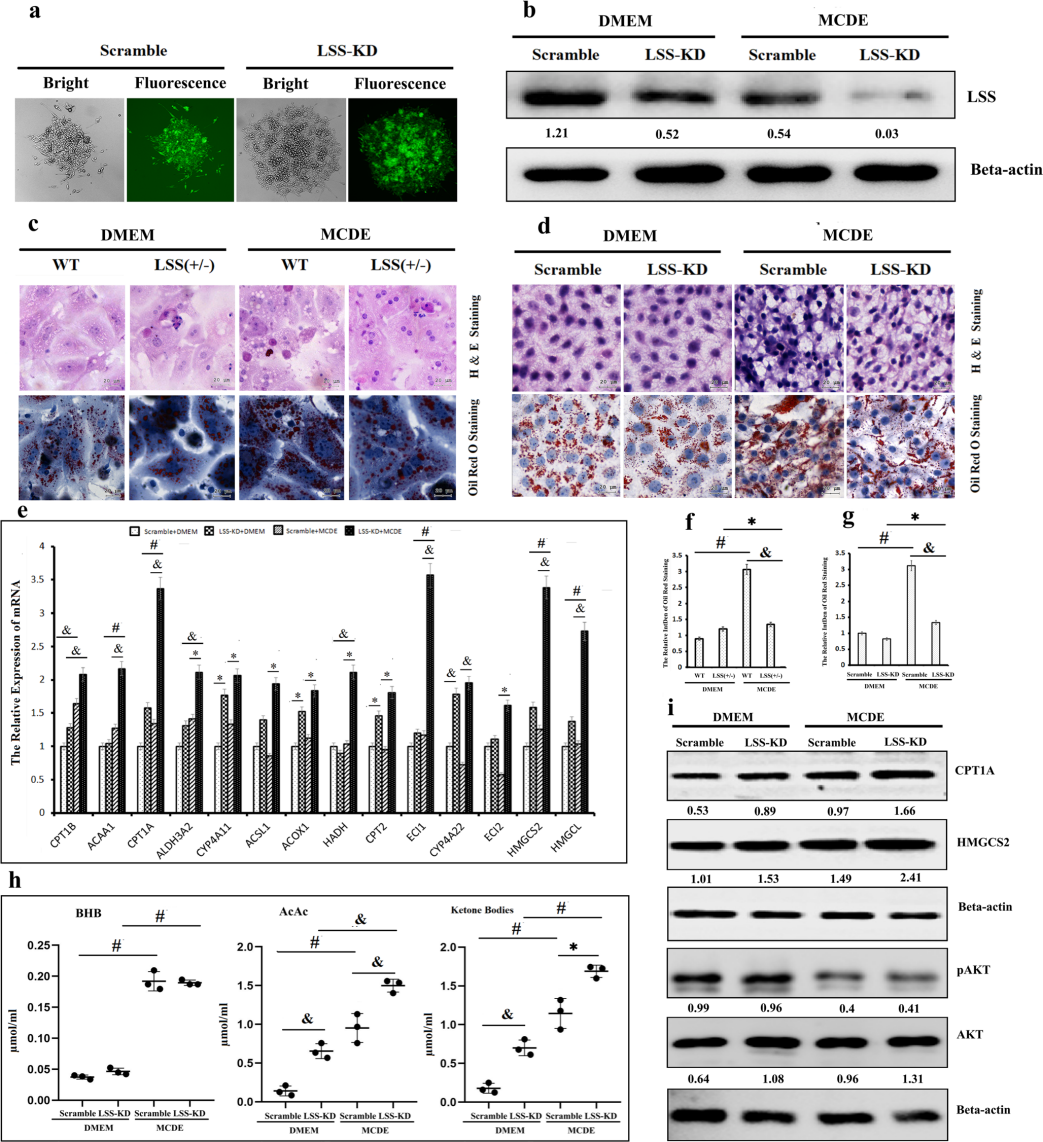
LSS loss of function alleviated MCDE induced cellular steatosis by activating β-oxidation and ketonebodies synthesis. (**a**) LSS shRNA transfection efficiency detected using GFP reporter, (**b**) LSS knockdown efficiency detected using Western blot analysis, (**c**) H&E and Oil Red O staining of primary hepatocytes isolated from ND and MCD-fed WT and LSS^+/−^ mice, (**d**) H&E and Oil Red O staining of DMEM and MCDE cultured HepG2 cells transfected with LSS shRNA or sramble shRNA, (**e**) mRNA levels of interested genes involved in ketogenesis and fatty acid catabolism in DMEM and MCDE cultured HepG2 cells transfected with LSS shRNA or sramble shRNA, (**f**) Quantification of Oil Red O staining of primary hepatocytes isolated from ND and MCD-fed WT and LSS^+/−^ mice, (**g**) Quantification of Oil Red O staining of DMEM and MCDE cultured HepG2 cells transfected with LSS shRNA or sramble shRNA, (**h**) Ketone bodies levels in DMEM and MCDE cultured HepG2 cells transfected with LSS shRNA or sramble shRNA, (**i**) Protein levels of fatty acid β-oxidation and ketogenesis key enzymes in DMEM and MCDE cultured HepG2 cells transfected with LSS shRNA or sramble shRNA. ^*^p < 0.05, ^&^*p* < 0.01, ^#^*p* < 0.001

To further determine the possible mechanisms of LSS on MASLD development and confirm the findings gained in animal models, HepG2 cells with effective LSS knockdown (Fig. 6a and b) and primary hepatocytes isolated from LSS+/- mice or WT mice fed MCD diet were used to construct MCD-induced cell models. H&E and Oil Red O staining demonstrated the occurrence of steatosis under culture with MCDE (Fig. 6c and d) which means that MCDE efficiently induces steatosis in vitro. LSS shRNA transfected HepG2 cells and primary hepatocytes isolated from LSS+/- mice showed less vacuolar degeneration and smaller lipid droplets in response to MCDE (Fig. 6c and d). And also, elevated ketone bodies levels were dedected in MCDE cultured cells with LSS KD (Fig. 6g). Corresponding genes involved in ketogenesis and fatty acid degradation were changed similarly as in animal models analysed by qRT-PCR (Fig. 6e). Western blot assay showed that HMGCS2 and CTP1A were upregulated in MCDE cultured cells with LSS KD (Fig. 6f). On the whole, the results from cell models evidenced corroboratively that MCD- induced MASLD development was alleviated by promoting fatty acid degradation when LSS was inhibited.

### LSS is lower expressed in Livers of Human Subjects with MASLD and MCD induced mouse models of MASLD

**Fig. 7:**
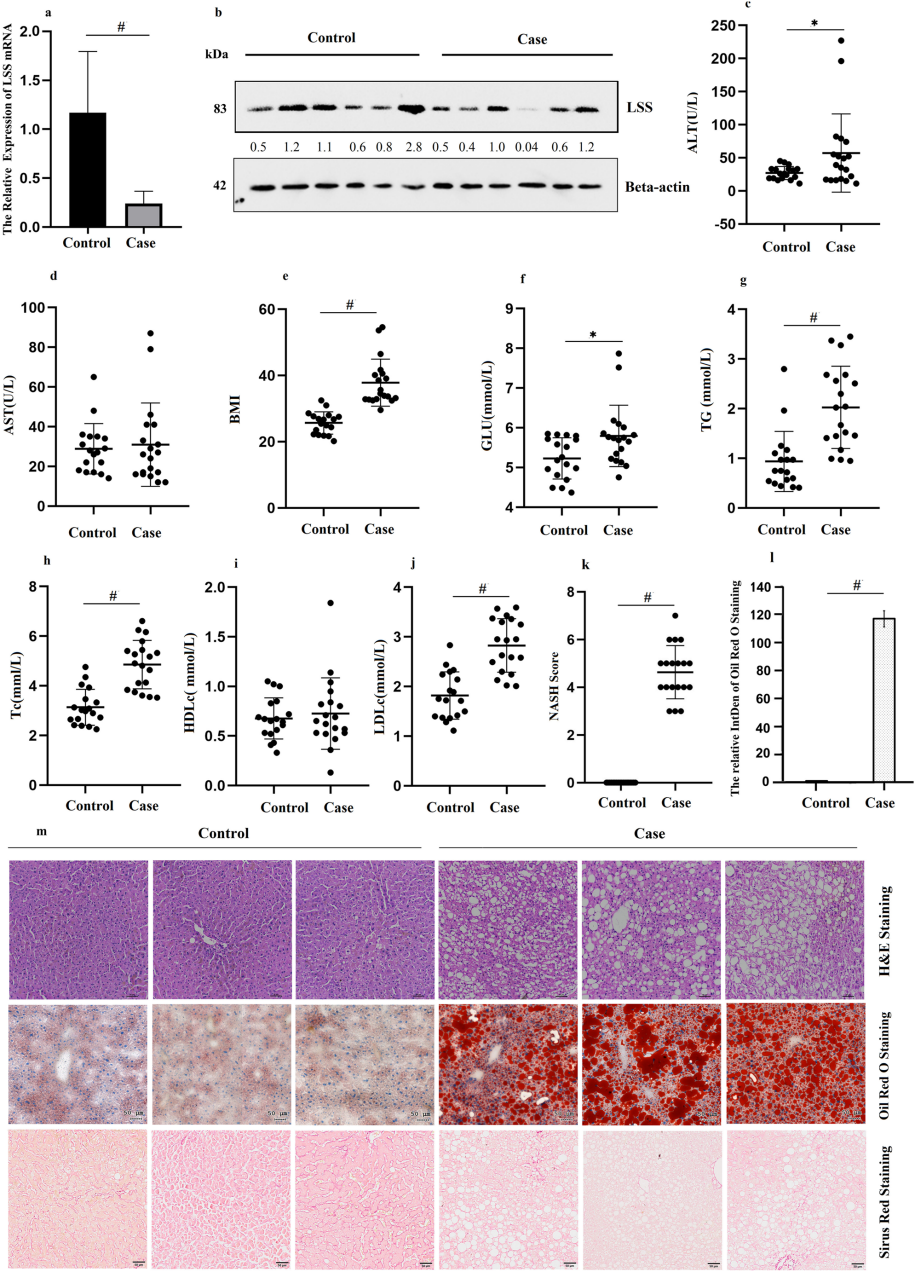
LSS was down-regulated in human MASLD patients. (**a**) LSS mRNA, and (**b**) LSS protein levels in livers of MASLD cases and control. (**c**) Plasma ALT, (**d**) Plasma AST, (**e**) BMI, (**f**) Fasting blood-glucose, (**g**) Serum TG, (**h**) Tc, (**i**) HDLc, (**j**) LDLc, (**k**) NASH score, (**l**) Quantification of Oil Red O staining and (**m**) Liver H&E, Oil Red O staing and Sirius Red staining of MASLD cases and control. *N* = 18 for MASLD patiets and *N* = 19 for control. ^*^p < 0.05, ^#^*p* < 0.001

The clinical significance of LSS in patients with MASLD was assessed according to the protective role of targeting LSS in MASLD mice models and cell models. Histological characteristics of MASLD with hepatic steatosis were shown by serum ALT level (Fig. 7c), H&E staining and Oil Red O staining, but without obvious hepatic fibrosis demonstrated by Sirius Red staining (Fig. 7l) [13]. Above histopathological features confirmed that the cases involved in our study are in the pathological stage of simple steatosis or steatohepatitis but no fibrosis has occurred. When LSS expression was examined in confirmed liver tissues, both mRNA and protein levels were found to be decreased in MASLD patients (Fig. 7a and b). Consistent with findings in clinical MASLD cases, LSS was found decreased in both MCD fed WT mice livers and MCDE cultured WT cells compared to ND fed and DMEM cultured ones, as shown in Figs. 2k and 6b. It was in line with some reports which revealed that elevated cholesterol levels of MASLD patients and mice caused a marked compensatory decrease in cholesterogenic gene expression such as LSS [24]. This was likely an adaptive change to disturbed cholesterol homeostasis which acts as a feedback inhibition on synthases to maintain cholesterol balance across the whole body in the intact animal. It can be speculated as one mechanism that organism tried to allow whole body cholesterol content to remain normal, both in human and mice MASLD cases.

## Discussion

MASLD is an umbrella term that encompasses a disease spectrum The pathological course of MASLD progresses from hepatic steatosis to MASH. If not treated timely, some patients gradually develop fibrosis, cirrhosis and eventually hepatocellular carcinomas [25]. The more sedentary lifestyle and a diet that is rich insaturated fats, sucrose and fructose in recent decades lead to a precipitous increase in MASLD [26]. Due to complex pathogenesis and unknown mechanisms of MASLD, a few therapies for MASLD and MASH were proven effective clinically [3]. Hence, it has become one of the most important and grim tasks of studies on MASLD to explore the molecular mechanisms of MASLD development in order to find new preventive strategies and therapeutic targets to manage these rampant hepatic diseases.

Epidemiological studies have found a positive association between cholesterol and MASLD [27]. There were experimental evidences that the level of free cholesterol was higher in the liver of MASLD patients [28]. High fat diet with cholesterol supplement in mice aggravated the development from simple hepatocyte steatosis to MASH [29]. These suggest that the increased cholesterol level may be one of the core causes of MASLD.

Endogenous cholesterol is achieved through a multistep enzymatic cascade. HMGCoA reductase catalyzes the rate-limiting step of the pathway, the conversion of HMG-CoA to mevalonate, has been developed as a target for clinical treatment of MASLD by inhibitor known as statins. Because the enzyme is located upstream of cholesterol anabolism, the inhibition of this enzyme leads to the reduction of a variety of downstream bioactive molecules (such as FPP, GGPP, CoQ10, etc.) and thus causes great side effects and is also hepatotoxic, although statins can improve MASLD [30]. Our preliminary observation in the livers of MASLD patients revealed a lower LSS expression than normal liver tissues. Based on our initial results, LSS may be involved in the pathological process of MASLD. LSS catalyzes the cyclization of epoxy squalene into the first sterol intermediate product (lanosterol) which is a critical step downstream of HMG-CoA reductase. When inhibiting the enzyme activity of LSS, the products of upstream active products are not affected with mainly decreased sterol products and cholesterol [31]. And also, inhibition of LSS could have the potential to stimulate cholesterol efflux from peripheral tissues by producing 24(S), 25-epoxycholesterol to activate LXR pathway and thus induce genes expression involved in reverse cholesterol transport and HDL formation [7].

Using LSS^+/−^ mice and also LSS inhibitor, RO48-8071, we observed apparently protective effects of targeting LSS on MCD -induced hepatic steatosis and injury by liver histology and serum biochemistry analysis. Targeting LSS alleviated vacuolar degeneration and lipid droplets formation in MASLD cell models.

LSS loss of function regulates hepatic cholesterol and triglyceride homeostasis through a complex set of mechanisms including lipid biosynthesis and catabolism. Transcriptomic analysis and KEGG pathway enrichment revealed a variety of gene expression changes involved in multiple metabolic pathways including fat acid β-oxidation, ketone bodies synthesis and so on. Impaired β-oxidation due to mitochondrial dysfunction in the livers with MASLD led to accumulation of TGs which could form lipid droplets and may also contribute to metabolic abnormalities in hepatocytes [23] and to drive progression of MASLD [32]. In our case, there is lower TG level and elevated expression of enzymes catalyzing fatty acid β-oxidation and ketogenesis, CTP1A and HMGCS2, in livers of LSS^+/−^ mice fed MCD diet when compared to WT ones and less damaged mitochondria was shown in LSS^+/−^ mice. Together with the significantly increased serum ketone bodies levels in MCD fed mice, LSS inhibition translates into an induction of CTP1A and HMGCS2 expression. Fatty acids β-oxidation was promoted which means that more fatty acids was decomposed and thus less fatty acids was used to synthesize TG. And TG is more easily metabolized than deposited in the liver. The above maybe the strategy of reducing TG in hepatocytes of MASLD mice with LSS loss of function, consistent with the alleviated steatosis shown by histological alteration.

In conclusion, we found LSS as a potential target for MASLD treatment. Increased fatty acids β-oxidation and ketone bodies formation in hepatic cells maybe one of the outlets of accumulated TG in LSS^+/−^ mice. The above mechanisms may participate in alleviated liver injury and hepatic steatosis by targeting LSS in the pathological processes of MASLD.

## Supplementary Information

Below is the link to the electronic supplementary material.

Supplementary Material 1 (DOCX 1.99 MB)

## Data Availability

All data included in this study are available on request from the corresponding author, SQZ, upon reasonable request.

## References

[1] Rinella ME, Lazarus JV, Ratziu V, Francque SM, Sanyal AJ, Kanwal F et al (2023) A multisociety Delphi consensus statement on new fatty liver disease nomenclature. Hepatology 78(6):1966–1986. 10.1097/hep.0000000000000520 37363821 10.1097/HEP.0000000000000520 PMC10653297

[2] Riazi K, Azhari H, Charette JH, Underwood FE, King JA, Afshar EE et al (2022) The prevalence and incidence of NAFLD worldwide: a systematic review and meta-analysis. Lancet Gastroenterol Hepatol 7(9):851–861. 10.1016/s2468-1253(22)00165-0 35798021 10.1016/S2468-1253(22)00165-0

[3] Harrison SA, Bedossa P, Guy CD, Schattenberg JM, Loomba R, Taub R et al (2024) A phase 3, Randomized, controlled trial of Resmetirom in NASH with liver fibrosis. N Engl J Med 390(6):497–509 10.1056/NEJMoa2309000 38324483 10.1056/NEJMoa2309000

[4] Friedman SL, Neuschwander-Tetri BA, Rinella M, Sanyal AJ (2018) Mechanisms of NAFLD development and therapeutic strategies. Nat Med 24(7):908–922. 10.1038/s41591-018-0104-9PubMed PMID: WOS:000438187700017 29967350 10.1038/s41591-018-0104-9 PMC6553468

[5] Doumas M, Imprialos K, Dimakopoulou A, Stavropoulos K, Binas A, Athyros VG (2018) The role of Statins in the management of nonalcoholic fatty liver disease. Curr Pharm Design 24(38):4587–4592. 10.2174/1381612825666190117114305 10.2174/1381612825666190117114305 30652643

[6] Chang T-Y, Chang CCY, Ohgami N, Yamauchi Y (2006) Cholesterol sensing, trafficking, and esterification. Annu Rev Cell Dev Biol 22(1):129–57. 10.1146/annurev.cellbio.22.010305.104656 16753029 10.1146/annurev.cellbio.22.010305.104656

[7] Rowe AH, Argmann CA, Edwards JY, Sawyez CG, Morand OH, Hegele RA et al (2003) Enhanced synthesis of the oxysterol 24(S),25-epoxycholesterol in macrophages by inhibitors of 2,3-oxidosqualene:lanosterol cyclase: a novel mechanism for the Attenuation of foam cell formation. Circul Res 93(8):717–725 10.1161/01.Res.0000097606.43659.F4 10.1161/01.RES.0000097606.43659.F4 14512442

[8] Bathish B, Robertson H, Dillon JF, Dinkova-Kostova AT, Hayes JD (2022) Nonalcoholic steatohepatitis and mechanisms by which it is ameliorated by activation of the CNC-bZIP transcription factor Nrf2. Free Radic Biol Med 188:221–261. 10.1016/j.freeradbiomed.2022.06.226 35728768 10.1016/j.freeradbiomed.2022.06.226

[9] Kleiner DE, Brunt EM, Van Natta M, Behling C, Contos MJ, Cummings OW et al (2005) Design and validation of a histological scoring system for nonalcoholic fatty liver disease. Hepatology 41(6):1313–1321. 10.1002/hep.20701 15915461 10.1002/hep.20701

[10] Lv T, Fan X, He C, Zhu S, Xiong X, Yan W et al (2024) SLC7A11-ROS/αKG-AMPK axis regulates liver inflammation through mitophagy and impairs liver fibrosis and NASH progression. Redox Biol. 10.1016/j.redox.2024.103159 38642501 10.1016/j.redox.2024.103159 PMC11047786

[11] Shao YJ, Guan YT, Wang LR, Qiu ZW, Liu MZ, Chen YT et al (2014) CRISPR/Cas-mediated genome editing in the rat via direct injection of one-cell embryos. Nat Protoc 9(10):2493–512. 10.1038/nprot.2014.171 25255092 10.1038/nprot.2014.171

[13] Brown GT, Kleiner DE (2016) Histopathology of nonalcoholic fatty liver disease and nonalcoholic steatohepatitis. Metabolism 65(8):1080–6. 10.1016/j.metabol.2015.11.008 26775559 10.1016/j.metabol.2015.11.008 PMC4889547

[14] Marra F, Svegliati-Baroni G (2018) Lipotoxicity and the gut-liver axis in NASH pathogenesis. J Hepatol 68(2):280–95. 10.1016/j.jhep.2017.11.014 29154964 10.1016/j.jhep.2017.11.014

[16] van der Veen JN, Kennelly JP, Wan S, Vance JE, Vance DE, Jacobs RL (2017) The critical role of phosphatidylcholine and phosphatidylethanolamine metabolism in health and disease. Biochimica et Biophysica Acta (BBA) - Biomembranes 1859(9):1558–72. 10.1016/j.bbamem.2017.04.006 28411170 10.1016/j.bbamem.2017.04.006

[17] Perry RJ, Kim T, Zhang X-M, Lee H-Y, Pesta D, Popov VB et al (2013) Reversal of hypertriglyceridemia, fatty liver disease, and insulin resistance by a liver-targeted mitochondrial uncoupler. Cell Metab 18(5):740–8. 10.1016/j.cmet.2013.10.004 24206666 10.1016/j.cmet.2013.10.004 PMC4104686

[18] Hou T, Tian Y, Cao Z, Zhang J, Feng T, Tao W et al (2022) Cytoplasmic SIRT6-mediated ACSL5 deacetylation impedes nonalcoholic fatty liver disease by facilitating hepatic fatty acid oxidation. Mol Cell 82(21):4099. 10.1016/j.molcel.2022.09.018 36208627 10.1016/j.molcel.2022.09.018

[19] Hegardt FG (1999) Mitochondrial 3-hydroxy-3-methylglutaryl-CoA synthase: a control enzyme in ketogenesis. Biochem J 338(Pt 3):569–582. 10.1042/0264-6021:3380569PubMed PMID: MEDLINE:10051425 10051425 PMC1220089

[20] Eaton S (2002) Control of mitochondrial beta-oxidation flux. Prog Lipid Res 41(3):197–239. 10.1016/s0163-7827(01)00024-8 11814524 10.1016/s0163-7827(01)00024-8

[22] Vyas S, Zaganjor E, Haigis MC (2016) Mitochondria and cancer. Cell 166(3):555–566. 10.1016/j.cell.2016.07.002 27471965 10.1016/j.cell.2016.07.002 PMC5036969

[23] Sanyal AJ, Campbell-Sargent C, Mirshahi F, Rizzo WB, Contos MJ, Sterling RK et al (2001) Nonalcoholic steatohepatitis: association of insulin resistance and mitochondrial abnormalities. Gastroenterology 120(5):1183–1192. 10.1053/gast.2001.23256 11266382 10.1053/gast.2001.23256

[24] Lorbek G, Perse M, Horvat S, Bjorkhem I, Rozman D (2013) Sex differences in the hepatic cholesterol sensing mechanisms in mice. Molecules 18(9):11067–11085. 10.3390/molecules180911067 24025456 10.3390/molecules180911067 PMC6270450

[25] Cohen JC, Horton JD, Hobbs HH (2011) Human fatty liver disease: old questions and new insights. Science 332(6037):1519–1523. 10.1126/science.1204265 21700865 10.1126/science.1204265 PMC3229276

[26] de Wit NJW, Afman LA, Mensink M, Muller M (2012) Phenotyping the effect of diet on non-alcoholic fatty liver disease. J Hepatol 57(6):1370–1373. 10.1016/j.jhep.2012.07.003 22796155 10.1016/j.jhep.2012.07.003

[27] Ioannou GN (2016) The role of cholesterol in the pathogenesis of NASH. Trends Endocrinol Metab 27(2):84–95. 10.1016/j.tem.2015.11.008 26703097 10.1016/j.tem.2015.11.008

[28] Gan LT, Van Rooyen DM, Koina ME, McCuskey RS, Teoh NC, Farrell GC (2014) Hepatocyte free cholesterol lipotoxicity results from JNK1-mediated mitochondrial injury and is HMGB1 and TLR4-dependent. J Hepatol 61(6):1376–84. 10.1016/j.jhep.2014.07.024 25064435 10.1016/j.jhep.2014.07.024

[29] Savard C, Tartaglione EV, Kuver R, Haigh WG, Farrell GC, Subramanian S et al (2013) Synergistic interaction of dietary cholesterol and dietary fat in inducing experimental steatohepatitis. Hepatology 57(1):81–92. 10.1002/hep.25789 22508243 10.1002/hep.25789 PMC5341743

[30] Diehl AM, Day C (2017) Cause, pathogenesis, and treatment of nonalcoholic steatohepatitis. N Engl J Med 377(21):2063–72. 10.1056/NEJMra1503519 29166236 10.1056/NEJMra1503519

[31] Thoma R, Schulz-Gasch T, D’Arcy B, Benz J, Aebi J, Dehmlow H et al (2004) Insight into steroid scaffold formation from the structure of human oxidosqualene cyclase. Nature 432(7013):118–22. 10.1038/nature02993 15525992 10.1038/nature02993

[32] Musso G, Cassader M, Paschetta E, Gambino R (2018) Bioactive lipid species and metabolic pathways in progression and resolution of nonalcoholic steatohepatitis. Gastroenterology 155(2):282. 10.1053/j.gastro.2018.06.031 29906416 10.1053/j.gastro.2018.06.031

