# Supplementary material for: Targeting lanosterol synthase alleviates MASLD by promoting fatty acid catabolism
Source: Cell Mol Life Sci. 2026 Mar 17;83(1):179. doi: 10.1007/s00018-026-06091-7 (PMC13038851; doi:10.1007/s00018-026-06091-7)
Supplement: Supplementary file 1 — Supplementary Material 1 (DOCX 1.99 MB) [file 18_2026_6091_MOESM1_ESM.docx]

**Supplementary Table 1.** **The primers used in this study**

| **Primers for Mouse** | | |
| --- | --- | --- |
| **Gene** | **Forward Primer Sequence** | **Reverse Primer Sequence** |
| LSS for gene types | ACCTGGGCGGAGTCTAAGGAAG | AAGGCTCTCCACTGTTTCAGAGCT |
| LSSfor gene expression | GGGGACCCTATAAAACTGA | GGGTCGTCCTCCGCTTGAT |
| Beta-actin | GCCACTGTCGAGTCGCGT | GATACCTCTCTTGCTCTGGGC |
| ACAA1A | CGCATCGCCCAATTTCTGAG | TTCTCCGAGGTCATCCCCAT |
| ALDH3A2 | GTACTGTGCTCCCTCACCTG | TCGCAGAAGCCAATTCAGGA |
| ACSL1 | TTTCTGATTCTGCTGCGGTG | ACCATCAGTGGTACCCGCTA |
| ACOX1 | TTTGTGGAACCTGTTGGCCT | AGGCCACCACTTGATGGAAG |
| CYP4A32 | CACTTTCCCTGATGGACGCT | AACCCTGGTAGGATCTGGCA |
| CYP4A10 | CCACAGGCAATGGCTACTCA | GAAAGGCACTTGGGAAGTTCT |
| CPT1B | AACACTACACGCATCCCAGG | TGAAGAAGGTCTGACGTGCC |
| CPT1A | GACTCCGCTCGCTCATTCC | GGCAGATCTGTTTGAGGGCT |
| CPT2 | TGATGGCTGAGTGCTCCAAAT | AACAGCATACGCAATGCCAA |
| ECI1 | GAGGTGTCATCCTCACGTCG | AACCAGAAGGGGGCAACAAT |
| ECI2 | CTGGTGTCCGAGTGTCCTG | AGTTTCCTTGGGTAGGCTGC |
| HADH | CAACCCCGTGCCTATGATGA | GTATGGCACCAAGAGTCGGT |
| HMGCS2 | AGAGGCCTTCAGGGGTCTAA | GGTAAAGGGAGGCCTTGGTC |
| HMGCL | AGGCTTTGAGGAAGCGGTAG | GGGAGACCTTCCCCTCGTAG |
| **Primers for Human** | | |
| LSS | CGGAGGGCACGTGTCTG | GCAGCCCCACGTAAAATGTC |
| Beta-actin | CCTCGCCTTTGCCGATCC | CCATCACGCCCTGGTGC |
| ACAA1 | TTAACTCCGCGGTCAGTTCC | AGTCTCCGGGATGTCACTCA |
| ACSL1 | GGTCCACGGAGGAGAATTCAG | GGTGGTGAGTGCTGCAAAAG |
| ACOX1 | TTACACACATCCTGGACGGC | GAAGTCTTTCCAAGCCCACC |
| ALDH3A2 | GGGTTGACGGTGGAGACAC | ACACATTGAATTCACTCTTGCACA |
| CYP4A11 | TGGGGAGATCAGACCCGAAA | AAAGGCATTCCTCACACGGG |
| CPT1A | CCAGACGAAGAACGTGGTCA | CCACCAGTCGCTCACGTAAT |
| CPT1B | GAGTGAACCCGAGCTGTGC | CAGGCGTTTCTTCCAGGAGT |
| CPT2 | AAGAAGCAGCAATGGGCCAG | AGGGTCCAGGTAGAGCTCAG |
| ECI1 | ATCGCCCCTTTCTGGTTGAAA | CTTGGTCAGCTGTCGAGCAT |
| ECI2 | TTCGTGTCCGAGTTCTCTGC | AGGCTCCACCTGACTAGAGG |
| CYP4A22 | CTCCGGGATCCTCCAAGTGA | CCATGGGATTTCGGGTCTGA |
| HADH | AGACAAGACCGATTCGCTGG | CACACGTTTGGAAGTCTCGTT |
| HMGCS2 | CCAGTAGCCCACCAAAGGTT | CCATGGCATAACGACCATCC |
| HMGCL | TATTTCTGTGCGGGGGTACG | TTTCATGATCCCTGGGGTGC |

**Supplementary Table 2.** **The antibodies used in this study**

| **Antibody** | **Supplier** | **Identifier** | **Application** | **Dilution** |
| --- | --- | --- | --- | --- |
| **Primary antibody** | | | | |
| LSS | Santa Cruz | sc-514507 | Western blot | 1:400 |
| CTP1A | Abcam | ab128568 | Western blot | 1:500 |
| HMGCS2 | Thermo Fisher Scientific | **PA5-55620** | Western blot | 1:1000 |
| pAKT | Cell Signaling Technology | 4060 | Western blot | 1:1000 |
| AKT | Cell Signaling Technology | 4691 | Western blot | 1:1000 |
| Beta-actin | Cell Signaling Technology | 3700 | Western blot | 1:1000 |
| Secondary antibody | | | | |
| Anti-mouse IgG, HRP | Thermo Fisher Scientific | 31430 | Western blot | 1:10000-1:100000 |
| Anti-rabbit IgG, HRP | Thermo Fisher Scientific | 31460 | Western blot | 1:10000-1:100000 |

**Supplementary Table 3. MCD Diet Composition**

|  | **MCD** | |
| --- | --- | --- |
|  | **gm** | **kcal** |
| Protein | 17 | 16 |
| Total Carbohydrate | 66 | 63 |
| Total Fat | 10 | 21 |
|  |  | 100 |
|  | 42 |  |
| Ingredient (gm) |  |  |
| L-Alanine | 3.5 | 14 |
| L-Arginine | 12.1 | 48.4 |
| L-Asparagine-H2O | 6 | 24 |
| L-Aspartate | 3.5 | 14 |
| L-Cystine | 3.5 | 14 |
| L-Glutamine | 40 | 160 |
| Glycine | 23.3 | 93.2 |
| L-Histidine-HCl-H2O | 4.5 | 18 |
| L-Isoleucine | 8.2 | 38.2 |
| L-Leucine | 11.1 | 44.4 |
| L-Lysine-HCl | 18 | 72 |
| L-Phenylalanine | 7.5 | 30 |
| L-Proline | 3.5 | 14 |
| L-Serine | 3.5 | 14 |
| L-Threonine | 8.2 | 38.2 |
| L-Tryptophan | 1.8 | 7.2 |
| L-Tyrosine | 5 | 20 |
| L-Valine | 8.2 | 38.2 |
| Total L-Amino Acids | 171.4 | 685.6 |
| Sucrose | 455.3 | 1821.2 |
| Corn Starch | 150 | 600 |
| Maltodextrin 10 | 50 | 200 |
| Cellulose | 30 | 0 |
| Corn Oil | 100 | 900 |
| Mineral mixture S10001 | 35 | 0 |
| Sodium Bicarbonate | 7.5 | 0 |
| Vitamin Mix V10001 | 10 | 40 |
| L-Methionine | 0 | 0 |
| Choline Bitrartate | 0 | 0 |
| FD&C Red Dye #40 | 0.05 | 0 |
| Total | 1009.25 | 4247 |

**Supplementary Table 4. Detailed characteristics of the MASLD patients and controls**

| **MASLD Cases** | | | | | | | | |
| --- | --- | --- | --- | --- | --- | --- | --- | --- |
|  | Gender | Age | Hight(m) | Weight(kg) | BMI | Drinking history | HBV | Scoring of the liver tissue |
| 1 | Male | 50 | 1.7 | 85.5 | 29.58477509 | non | non | 5 |
| 2 | Female | 40 | 1.68 | 95 | 33.65929705 | non | non | 5 |
| 3 | Female | 26 | 1.62 | 87 | 33.15043439 | non | non | 6 |
| 4 | Male | 26 | 1.78 | 122 | 38.50523924 | non | non | 4 |
| 5 | Male | 19 | 1.8 | 135 | 41.66666667 | non | non | 4 |
| 6 | Female | 38 | 1.63 | 145 | 54.5748805 | non | non | 5 |
| 7 | Female | 21 | 1.65 | 97 | 35.62901745 | non | non | 6 |
| 8 | Female | 34 | 1.6 | 84 | 32.8125 | non | non | 6 |
| 9 | Female | 33 | 1.58 | 81 | 32.44672328 | non | non | 7 |
| 10 | Male | 21 | 1.8 | 130 | 40.12345679 | non | non | 3 |
| 11 | Female | 46 | 1.55 | 78 | 32.46618106 | non | non | 4 |
| 12 | Female | 26 | 1.63 | 90 | 33.87406376 | non | non | 3 |
| 13 | Male | 20 | 1.7 | 155 | 53.63321799 | non | non | 5 |
| 14 | Male | 23 | 1.72 | 120 | 40.5624662 | non | non | 5 |
| 15 | Female | 29 | 1.55 | 83 | 34.54734651 | non | non | 4 |
| 16 | Female | 30 | 1.69 | 93.5 | 32.73694899 | non | non | 4 |
| 17 | Male | 34 | 1.64 | 125 | 46.47531231 | non | non | 4 |
| 18 | Female | 44 | 1.55 | 79 | 32.88241415 | non | non | 5 |
| 19 | Male | 28 | 1.6 | 100 | 39.0625 | non | non | 3 |
| **Healthy controls** | | | | | | | | |
|  | Gender | Age | Hight(m) | Weight(kg) | BMI | Drinking history | HBV | Scoring of the liver tissue |
| 1 | Male | 44 | 1.77 | 67 | 21.38593635 | non | non | 0 |
| 2 | Female | 35 | 1.59 | 53 | 20.96436059 | non | non | 0 |
| 3 | Male | 19 | 1.73 | 67 | 22.38631428 | non | non | 0 |
| 4 | Female | 24 | 1.62 | 56 | 21.33821064 | non | non | 0 |
| 5 | Male | 44 | 1.79 | 73 | 22.78330889 | non | non | 0 |
| 6 | Male | 28 | 1.69 | 62 | 21.70792339 | non | non | 0 |
| 7 | Female | 31 | 1.64 | 55.5 | 20.63503867 | non | non | 0 |
| 8 | Female | 24 | 1.63 | 56 | 21.07719523 | non | non | 0 |
| 9 | Female | 50 | 1.57 | 51 | 20.69049454 | non | non | 0 |
| 10 | Male | 38 | 1.74 | 68.5 | 22.62518166 | non | non | 0 |
| 11 | Male | 49 | 1.65 | 63 | 23.14049587 | non | non | 0 |
| 12 | Male | 40 | 1.76 | 72 | 23.24380165 | non | non | 0 |
| 13 | Female | 37 | 1.6 | 56.5 | 22.0703125 | non | non | 0 |
| 14 | Male | 34 | 1.71 | 64 | 21.88707637 | non | non | 0 |
| 15 | Male | 47 | 1.7 | 68 | 23.52941176 | non | non | 0 |
| 16 | Female | 28 | 1.69 | 61.5 | 21.53285949 | non | non | 0 |
| 17 | Female | 38 | 1.61 | 57 | 21.98989237 | non | non | 0 |
| 18 | Female | 24 | 1.59 | 53 | 20.96436059 | non | non | 0 |

**Figure legends of Supplementary Figures**

**Supplementary Figure 1.** (a) Livers of ND and MCD-fed WT and LSS^+/-^ mice, (b) Livers of ND and MCD-fed WT mice with or without RO 48-8071 injection, (c) Food intake/body weight/day, (d) Body weight and (e) Liver index of WT and LSS^+/-^ mice fed ND and MCD ND. (f) Food intake/body weight/day, (g) Body weight and (h) Liver index of MCD fed WT mice with or without RO 48-8071 injection. Serum levels of (i) TG, (j) Tc, (k) HDLc, (l) LDLc and (m) GLU of WT and LSS^+/-^ mice fed ND and MCD. Serum levels of (n) TG, (o) Tc, (p) HDLc, (q) LDLc and (r) GLU of ND and MCD fed WT mice with or without RO 48-8071 injection. N=10 mice for each group. ^*^*p*<0.05, ^&^*p*<0.01, ^#^*p*<0.001.

**Supplementary Figure 2.** Sirius Red staining of the livers.

**Supplementary Figures**

**
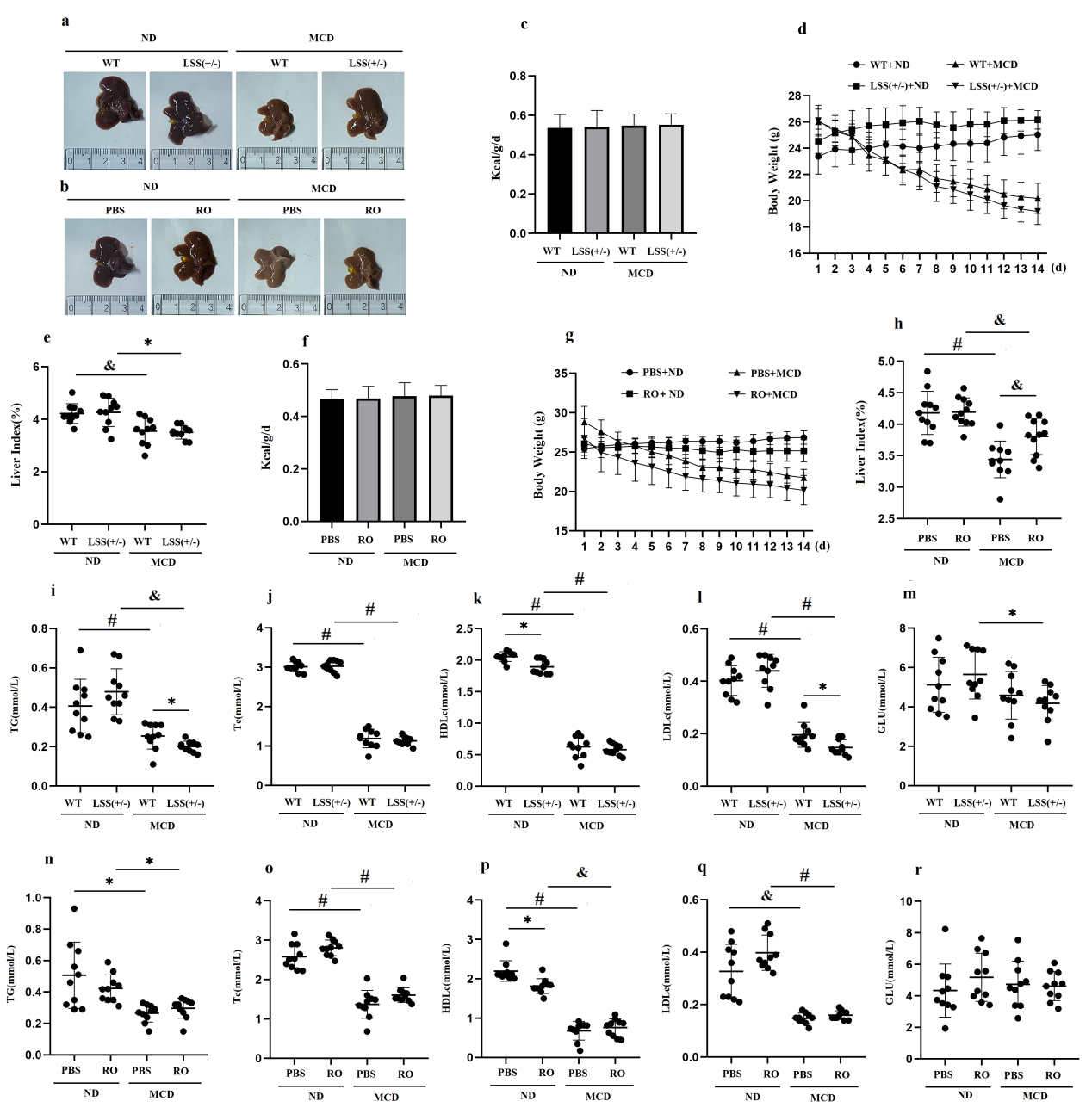
**

**Supplementary Figure 1.**


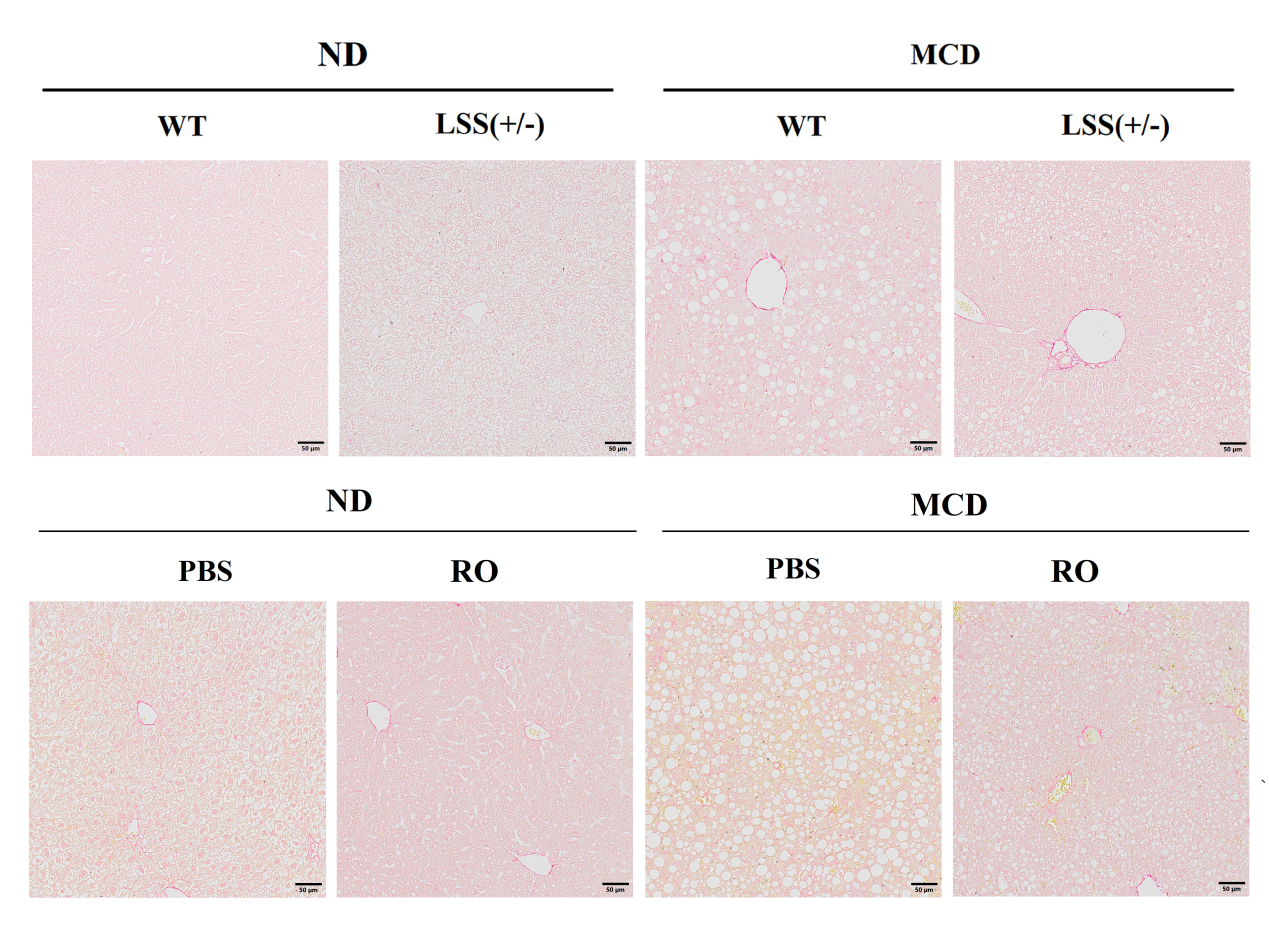


**Supplementary Figure 2.**

**Supplementary Materials and Methods**

Experimental materials and reagents

Normal chow diet (ND, D11112201, New Brunswick, NJ, USA) and methionine and choline-deficient (MCD) diet was from Research Diets ([A02082002BR](https://researchdiets.com/formulas/A02082002BR), New Brunswick, NJ, USA). High fat diet (HFD) with fat function ratio of 60% was customized from Boaigang Biotechnology (Boaigang12492M, Beijing, China). MG132 was from Sigma Aldrich (M8699, St Louis, MO, USA) and dissolved in DMSO ([D8418](https://www.sigmaaldrich.cn/CN/zh/product/sigma/d8418), Sigma Aldrich, St Louis, MO, USA) to prepare a stock solution at 1.5 mM concentration. Tunicamycin (TM, ab120296, Abcam, Cambridge, UK) was purchased from Abcam and stock solutions at 7.5 mg/ml were prepared by dissolution in DMSO. RO 48‑8071 was purchased from MedChemExpress (HY‑18630A, NJ, USA) and dissolved in ddH_2_O with a storage concentration of 10 mM. Working solutions of RO were prepared by further dissolution in sterile NS. Dulbecco's Modified Eagle Medium (DMEM) was purchased from Gibco (2463414; Thermo Fisher Scientific, Waltham, MA, USA), while Fetal Bovine Serum (FBS) was obtained from Clark Bioscience (FB25015, Clark Bioscience, VA, USA). Reagents used for biochemical analyze are from Wako Pure Chemical Industries (Osaka, Japan). The antibodies used in the present study are listed in Supplementary Table 2. BCA protein assay kit was from Pierce (23227, Thermo Fisher Scientific, Waltham, MA, USA). [SuperSignal West](https://www.thermofisher.cn/order/catalog/product/A38554) ECL kit was purchased from Thermo Fisher Scientific (34094, Thermo Fisher Scientific, Waltham, MA, USA). Oil Red O ([O1391](https://www.sigmaaldrich.cn/CN/zh/product/sigma/o1391)) and Sirius Red ([365548](https://www.sigmaaldrich.cn/CN/zh/product/sial/365548)) were from Sigma-Aldrich.

**Ethics statement**

All patients involved in the study have provided informed consent for using the samples for research purposes after approved by the Institutional Review Board (IRB) with approval no. 20200288. All mouse studies and experimental procedures were conducted after approved by Institutional Animal Care and Use Committee of Anhui Medical University (Hefei, Anhui, China, permits 20201102). In conducting research with mice, the investigators adhered to the strict guidelines and regulations of Institutional Animal Ethics Committee in Anhui Medical University. The mice were maintained in the temperature (22±2 ℃) and humidity (55±5 %) controlled specific‑pathogen‑free laboratory animal room under humane conditions with food and water provided adlibitum. Animal health and behavior were monitored daily by food and water intake, general assessment of animal activity and fur condition.

**Human samples**

The liver tissues of MASLD patiets (n=19) were collected at the First Affiliated Hospital of Anhui Medical University. Liver tissues of healthy controls were from the patients (n=18) undergoing hepatic hemangioma resection with matched age and gender with MASLD patients involved in the present study. Liver histology by H&E staining was scored by pathologists according to the Kleiner scoring system {Shao, 2014 #82}[1]. Cases were classified as MASLD with a MASLD activity score ≥3 and were treated as normal controls with MASLD activity score of 0. Individuals with viral infection (hepatitis B or C virus), drug or toxin use, excessive alcohol consumption (for men, >140 g/week; for women, >70 g/week) or other decompensated liver diseases such as liver autoimmune were excluded from the present study [2].

**Generation of LSS knockout mice**

LSS^+/-^ mice were prepared by Biomedical Research Institute of Nanjing University (Nanjing, China) using a pure C57BL/6 background. Global LSS knockout mice were acquired via CRISPR/Cas9 system [3]. Cas9 mRNA and sgRNA (5’-GAGCCGCCAGCGGGTGAGAT-3’ followed by the PAM sequence CGG) targeting LSS gene were co-injected into zygotes. sgRNA directed Cas9 endonuclease cleavage at exon 2 and created a double-strand break. Such breaks were repaired by non-homologous end joining and resulted in disruption of LSS gene with 46 bps deletion by frame shift from exon 2. The microinjected zygotes were transplanted into pseudopregnant mice. The genomic DNA of the newborn F0 mice was extracted for sequencing using primers flanking the target site. Transgenic positive founder mice were mated with wild-type mice to generate positive F1 mice and the pups were genotyped by PCR followed by sequence analysis. Knockout and WT genotypes were verified with PCR analysis of tail clips using the primers forward ACCTGGGCGGAGTCTAAGGAAG and reverse AAGGCTCTCCACTGTTTCAGAGCT.

**Methionine and choline-deficient diet model [2]**

LSS heterozygous knockout C57BL/6 mice (Homozygous knockout of LSS gene is lethal on the 9th day of embryonic stage) which were referred as LSS^+/-^ hereinafter and their non-transgenic littermates (WT) were fed ND or MCD diet for two weeks at 8 weeks of age. The groups are as follow (n=10 for each group): ND-fed WT mice, MCD-fed WT mice, ND-fed LSS^+/-^ mice, and MCD-fed LSS^+/-^ mice.

RO 48‑8071, an inhibitor of LSS, was used to confirm the role of LSS loss of function in MCD-induced liver injury model. Male eight-week-old WT mice were feed with ND or MCD diet (n=10 for each group) for two weeks, with or without intraperitoneal injection of 100 µl fresh prepared RO 48‑8071 in PBS at 10 mg/kg/day, with PBS serving as control.

**Construction of LSS knockdown cell line**

HepG2 cells, a line of human hepatocytes, were kindly gifted by Professor Huabing Zhang (Anhui Medical University, Hefei, Anhui, China) and cultured in DMEM containing 10 % FBS, 100 U/mL penicillin and 100 μg/mL streptomycin (15070063, Thermo Fisher Scientific, Waltham, MA, USA), in a humidified incubator at 37 °C with 5 % CO_2_.

Cells were transfected with confirmed shLSS-pRNAT-U6.1/Neo targeting human LSS gene sequences GGACTGCGCTCAACTATGT or pRNAT-U6.1/Neo vector plasmids using Lipofectamine 2000 (11668019, Thermo Fisher Scientific, Waltham, MA, USA) and selected with G418 (HY-17561, MedChemExpress, NJ, USA) to get stably transfected HepG2 cell clones. The efficiency of transfection and RNA interference by shRNAs were verified by green fluorescence and LSS expression level, respectively.

**Methionine and choline-deficient cell model**

HepG2 cells with LSS knockdown attaining 70-80% confluence were exposed to identical medium that was manufactured to be completely deficient of methionine and choline (MCDE, Thermo Fisher Scientific, Waltham, MA) for 48 h to establish the cellular model. And also the primary hepotocytes were enzymatically isolated from mice livers of LSS^+/-^ and WT mice fed MCD diet. The above two kinds of model cells grown on coverslips in 12-well plates (Corning, NY, USA) were stained by TPE-MI to visualize unfolded protein, or Oil Red O to detect fat accumulation, or fixed for H&E staining. The cells in 6-well plates (Corning, NY, USA) were harvested for Western blot analyses.

**Biochemistry analysis**

The plasma levels of Alanine aminotransferase (ALT), aspartateaminotransferase (AST), triglyceride (TG), total cholesterol(TC) , HDL-C, LDL-C and glucose (GLU) were measured using HITACHI 3100 automatic biochemical analyzer (HITACHI3100, Tokyo, Japan) according to the manufacturer's instructions.

**Analysis of lipid components in the liver**

Extracts of liver tissues, made according to the instruction of the enzymatic kit (E1025, GPO-POD, Applygen Technologies, Beijing, China), were used on the measurements of TG, Tc, HDLc and LDLc. Briefly, frozen livers were weighted, homogenized in lysis solution (20 μL/mg tissue) and incubated at 70 °C for 10 min. The samples were centrifuged for 5 min at 2,000 rpm at room temperature. The supernatants were collected to measure concentrations of TG, Tc, HDLc and LDLc in livers using HITACHI 3100 automatic biochemical analyzer.

Lipidomic Profiling of liver tissues was performed using ultra-high performance liquid chromatography (UHPLC) Dionex Ultimate 3000 (Thermo Fisher Scientific, San Jose, USA) system equipped with SRD-3600 degasser, HPG-3400RS binary pump, WPS-3000TRS autosampler and TCC-3000RS column oven to observe the differences in lipid metabolites among the groups. The UHPLC chromatographic system was coupled to a Q-Exactive Plus Hybrid Quadrupole Orbitrap High-resolution Mass Spectrometer (Thermo Fisher Scientific, San Jose, CA). Briefly, 20 mg liver tissue was homogenized in cold methanol/water (3:1, v/v) with weight ratio of 1:10 and extracted by 600 μL pre-cooled methanol/dichloromethane (1:2, v/v) solvent. Add 200 μL water and mix well before centrifugation at 12000 rpm for 10 min at 4 °C. The subjacent organic layer was transferred to a new glass tube, and evaporated to dryness using an evaporator at 37 °C under a stream of nitrogen. The residue was reconstituted by 200 µL of solvent (isopropanol/methanol, 1:1, v/v), filtered through a 0.22 µm syringe filter and measured in positive and negative ion modes by non-targeted UPLC-MS/MS lipidomics technology. The collected raw data were processed by Lipid Search software (version 4.1, Thermo Fisher Scientifc, San Jose, USA) for chromatographic peak deconvolution, identification, alignment and integration. The compounds with peak area <20 % in the QC samples were 75.7 % and 85.7 %, respectively, indicating that the method is repeatable and reliable. The model was evaluated by 200 permutation inspection tests and the result (R^2^=0.559, Q^2^=-0.543) shows that the established OPLS-DA model is reliable. One-dimensional statistical analysis includes Student's t-test and multiple of variation analysis, combines VIP (Variable important in projection), Fold-change value and P value was used to screen out significantly different substances. Multivariable receiver operating characteristic (ROC) curve was used to analyze the differences of the substances in the table among the groups.

**Histology Staining**

**H&E staining for general observation**

Fresh liver tissues were fixed in 10 % formalin for 48 h, and then dehydrated and embedded in paraffin. Paraffin-embedded liver tissues were sliced into 4 μm sections (HistoCore AUTOCUT, Leica RM2255, Germany). The slices were dried and rehydrated by serial immersion in xylene and ethanol. The treated cells were fixed in 10 % formalin for 30 min at room temperature. H&E staining was performed using a standard protocol. The sections were incubated in hematoxylin for 5 min, washed with ddH_2_O for 15 min, and stained with eosin for 2 min. The sections were then dehydrated and mounted with a neutral resin onto slides.

**Oil Red O staining for intracellular lipid droplets observation**

Fresh liver tissues were snap frozen in liquid nitrogen and embedded in OCT embedding medium, and then sliced into 8 μm thick sections on a frozen section machine (Cryostat microtome, Leica CM3050S，Germany). The sections dried on slides or the treated cells were fixed in 10 % formalin for 30 min at room temperature and washed with PBS. To assess lipid accumulation, fixed sections or cells were stained with freshly diluted Oil Red O staining solution for 1 h after 5 min incubation in 60% isopropanol, washed thoroughly with 60% isopropanol for 3 times, and counterstained with hematoxylin for 2 min. The slides were then washed with ddH_2_O for 3 times, dried, and mounted with glycerogelatin. Lipid accumulation in liver tissues and cells was quantified via the amount of Oil Red O staining using Image J Software.

**Sirius Red staining for collagen fiber detection**

Paraffin-embedded histology slides were rehydrated and incubated with 0.05 % Sirius Red in saturated picric acid ([41506](https://www.sigmaaldrich.cn/CN/zh/product/sial/41506), Sigma Aldrich, St Louis, MO) for 30 min followed by serial immersion in 95 % ethanol, absolute ethanol and xylene. The mounted slides with neutral resin were pictured to observe collagen fibers.

All histological slides were visualized on TissueFAXS Plus (version 7.1, TissueGnostics GmbH, Vienna, Austria). Pathologic features of the liver sections were graded in a blinded fashion according to the Kleiner scoring system to assess the severity of MASLD [1]. The score was generated by adding the individual scores for the features of steatosis, ballooning and lobular inflammation. The average score of histological characteristic in each group was presented.

**Ultrastructural analysis by electronic microscopy**

For ultrastructural investigations of liver, fresh liver tissues of the mice were cut into small blocks with a volume of about 1 mm^3^ and immediately fixed with 3 % glutaraldehyde (prepared in 0.1 M phosphate buffer, pH 7.4) overnight at 4^°^C. After washed in phosphate buffer for three times, the blocks were post-fixated with 1 % osmium tetroxide (prepared in 0.1 M phosphate buffer, pH 7.4) for 30 minute at room temperature. Tissues were stepwise dehydrated and embedded in Epon 812 (18010, Ted Pella, CA). Ultrathin sections were cut at 70 nm and double-stained with uranyl acetate and lead citrate, and photographed by transmission electron microscope (Talos L120C G2, Thermo Fisher Scientific, Waltham, MA, USA).

**Quantitative transcriptome analysis of liver tissues**

Total RNA was extracted using TRIzol (15596018, Thermo Fisher Scientific, Waltham, MA, USA) from fresh liver tissues and mRNA was enriched using oligo (DT) magnetic beads. Purified mRNA was fragmented into small pieces with fragment buffer at appropriate temperature. The first strand of cDNA was synthesized by adding random primers to the fragmented mRNA followed by a second-strand cDNA synthesis. Afterwards, double-stranded cDNA fragments are subjected to end-repair and adaptor ligation. PCR was performed to amplify the cDNA above. The PCR products were denatured into a single strand and then cyclized to obtain a single strand circular DNA library. The final library was obtained after the non cyclized linear DNA molecules were digested. Single stranded circular DNA molecules were replicated through rolling rings to form DNA nanoball (DNB), which are loaded into patterned nanoarrays using high-intensity DNA nanochip technique and sequenced through combinatorial Probe-Anchor Synthesis (cPAS).

Quantification of gene expression levels and differential expression analysis were carried out as follows. Raw reads were first filtered using SOAPnuke software (v1.5.2) [4] by removing the reads containing the connector, or with unknown base N content greater than 5 %, or low-quality reads (reads with a mass value of less than 10 accounting for more than 20 % of the total base number of the reads were defined as low-quality reads). And the clean reads were saved as FASTQ format. The subsequent analysis and data mining were performed on Dr. Tom Multi-omics Data mining system (<https://biosys.bgi.com>). The clean reads were mapped to the reference genome using HISAT2 (v2.0.4) [5] and aligned to the reference coding gene set by Bowtie2 (v2.2.5) [6]. Expression level of gene was calculated by RSEM (v1.3.1) [7]. The heatmap was drawn by pheatmap (Raivo Kolde, v1.0.8) according to the gene expression in different samples. Essentially, differential expression analysis was performed using the DESeq2 (v1.4.5) [8] with Q value ≤ 0.05.

**Array data and pathway analysis**

GO (http://www.geneontology.org/) and Kyoto Encyclopedia of Genes and Genomes (KEGG, https://www.kegg.jp/) enrichment analysis of annotated different expression gene were performed by Phyper (<http://en.wikipedia.org/wiki/Hypergeometric_distribution>) based on Hypergeometric test. The significant levels of terms and pathways were corrected by Q value with a rigorous threshold (Q value ≤0.05) (<http://github.com/jdstorey/qvalue>).

**Validation of target genes using qRT-PCR analysis**

Quantitative real-time PCR (qRT-PCR) was done to evaluate and confirm their expression levels of interested genes which were shown differently expressed between groups in the array results. Snap frozen liver tissues were homogenized and cultured cells were harvested before being lysed in TRIzol to isolate total RNA according to the manufacturer’s instructions. Five hundred ng of total RNA was reversely transcribed to cDNA with a Primer-Script RT kit (RR037A; Takara Bio, Dalian, China). Two-step quantitative real-time PCR was performed using Thermal Cycler DiceR Real Time System (Takara Bio, Dalian, China) monitored by SYBR ExScript RT-PCR Kit (Takara Bio, Dalian, China). Melting curve analysis was employed to determine the specificity of amplification. Relative gene transcription level was calculated using ΔΔCT method with Beta-actin being the house-keeping gene. The primers used for target genes are listed in Supplementary Table 1.

**Western blot analysis**

Protein extracted from snap frozen liver tissues and treated cells with RIPA lysis buffer (P0013B, Beyotime, Shanghai, China) and complete protease inhibitor cocktail (04693132001; Roche, Basel, Switzerland) was quantified using BCA protein assay kit. Equal amounts of heat-denatured protein was loaded, subjected to SDS-PAGE and transferred onto polyvinylidene fluoride (PVDF) membrane (IPVH00010; Millipore, Billerica, MA, USA). After blocked in 5 % non-fat milk in TBST (0.1 % Tween 20 in Tris buffered saline (TBS)) at room temperature for 2 h, the membranes were probed with primary antibodies at 4 °C overnight. After being washed in TBST, the samples were incubated in horseradish peroxidase-conjugated secondary antibody at room temperature for 1 h. Chemiluminescence signals were developed by a chemiluminescent imaging instrument (14T12NPFLI6-348, Tanon, China) using an ECL kit. The grayscale of each objective band was determined using Image J Software and the densitometric analysis of all Western blots bands were shown as relative intensity to that of Beta-actin. Antibodies used are listed in Supplementary Table 2.

**Statistical Analysis**

All statistical tests were performed using SPSS and GraphPad Software in the present study. All the data were expressed as means ± Standard error of the mean (SEM). The significance of differences between values was determined using the unpaired Student t test to calculate 2-tailed *p* value. Threshold for statistical significance was *p*<0.05.

**References**

1. Kleiner DE, Brunt EM, Van Natta M, Behling C, Contos MJ, Cummings OW, et al. Design and validation of a histological scoring system for nonalcoholic fatty liver disease. Hepatology. 2005;41(6):1313-21. doi: 10.1002/hep.20701. PubMed PMID: WOS:000229517300014.

2. Lv T, Fan X, He C, Zhu S, Xiong X, Yan W, et al. SLC7A11-ROS/αKG-AMPK axis regulates liver inflammation through mitophagy and impairs liver fibrosis and NASH progression. Redox Biology. 2024;72. doi: 10.1016/j.redox.2024.103159. PubMed PMID: WOS:001233610400001.

3. Shao Y, Guan Y, Wang L, Qiu Z, Liu M, Chen Y, et al. CRISPR/Cas-mediated genome editing in the rat via direct injection of one-cell embryos. Nature Protocols. 2014;9(10):2493-512. doi: 10.1038/nprot.2014.171. PubMed PMID: WOS:000343227800016.

4. Li R, Li Y, Kristiansen K, Wang J. SOAP: short oligonucleotide alignment program. Bioinformatics. 2008;24(5):713-4. doi: 10.1093/bioinformatics/btn025. PubMed PMID: WOS:000253746400017.

5. Kim D, Langmead B, Salzberg SL. HISAT: a fast spliced aligner with low memory requirements. Nature Methods. 2015;12(4):357-U121. doi: 10.1038/nmeth.3317. PubMed PMID: WOS:000352083100026.

6. Langmead B, Salzberg SL. Fast gapped-read alignment with Bowtie 2. Nature Methods. 2012;9(4):357-U54. doi: 10.1038/nmeth.1923. PubMed PMID: WOS:000302218500017.

7. Li B, Dewey CN. RSEM: accurate transcript quantification from RNA-Seq data with or without a reference genome. Bmc Bioinformatics. 2011;12. doi: 10.1186/1471-2105-12-323. PubMed PMID: WOS:000294361700001.

8. Love MI, Huber W, Anders S. Moderated estimation of fold change and dispersion for RNA-seq data with DESeq2. Genome Biology. 2014;15(12). doi: 10.1186/s13059-014-0550-8. PubMed PMID: WOS:000346609500022.
